# Small Molecule-Based Prodrug Targeting Prostate Specific Membrane Antigen for the Treatment of Prostate Cancer

**DOI:** 10.3390/cancers13030417

**Published:** 2021-01-22

**Authors:** Xinning Wang, Aditi Shirke, Ethan Walker, Rongcan Sun, Gopolakrishnan Ramamurthy, Jing Wang, Lingpeng Shan, Joey Mangadlao, Zhipeng Dong, Jing Li, Ziying Wang, Mark Schluchter, Dong Luo, Yu Wang, Shaun Stauffer, Susann Brady-Kalnay, Christopher Hoimes, Zhenghong Lee, James P. Basilion

**Affiliations:** 1 Department of Biomedical Engineering, Case Western Reserve University, 11100 Euclid Ave, Wearn Building B-49, Cleveland, OH 44106, USA; aas151@case.edu (A.S.); yvv@case.edu (E.W.); 2 Department of Radiology, Case Western Reserve University, 11100 Euclid Ave, Wearn Building B-44, Cleveland, OH 44106, USA; rxs814@case.edu (R.S.); gxr25@case.edu (G.R.); jxw373@case.edu (J.W.); joeydmangadlao@gmail.com (J.M.); dxl576@case.edu (D.L.); wangyu.vivian@outlook.com (Y.W.); zxl11@case.edu (Z.L.); 3 Department of Population and Quantitative Health Sciences, Case Western Reserve University, 2103 Cornell Rd, Cleveland, OH 44106, USA; Lingpengshan@gmail.com (L.S.); mds11@case.edu (M.S.); 4 Department of Mechanical Engineering, Case Western Reserve University, 2190 Adelbert Rd, Cleveland, OH 44106, USA; zdong6@jhu.edu (Z.D.); zaw11@case.edu (Z.W.); 5 Department of Chemistry, Case Western Reserve University, 2080 Adelbert Rd, Cleveland, OH 44106, USA; 18793158842@163.com; 6 Center for Therapeutics Discovery, Cleveland Clinic, 9500 Euclid Ave, Cleveland, OH 44195, USA; stauffs2@ccf.org; 7 Department of Molecular Biology and Micro Biology, Case Western Reserve University, 2190 Adelbert Rd, Cleveland, OH 44106, USA; susann.brady-kalany@case.edu; 8 Duke Cancer Institute, School of Medicine, Duke University, 905 S LaSalle St, GSRB2, Durham, NC 27710, USA; christopher.hoimes@duke.edu

**Keywords:** prostate cancer, prostate specific membrane antigen (PSMA), prodrug, monomethyl auristatin E (MMAE)

## Abstract

**Simple Summary:**

Currently, there is no effective treatment that can cure metastatic prostate cancer. Various pros-tate specific membrane antigen (PSMA)-targeted radioimaging and radiotherapy agents have en-tered clinical trials; however, no PSMA-targeted chemotherapy is currently in clinical trials. We used a small molecular weight PSMA ligand and developed a new prodrug PSMA-1-VcMMAE. Although the linker and the payload drug monomethyl auristatin E (MMAE) have been widely used in antibody–drug conjugate (ADC) development, it has never been tried as a combination with a PSMA targeting ligand. Our PSMA-targeting drug conjugate showed potent and selective in vitro and in vivo antitumor activity with no toxicity observed. It has superior therapeutic index and will likely impact therapeutic management of lethal prostate cancers.

**Abstract:**

Metastatic castration-resistant prostate cancer poses a serious clinical problem with poor outcomes and remains a deadly disease. New targeted treatment options are urgently needed. PSMA is highly expressed in prostate cancer and has been an attractive biomarker for the treatment of prostate cancer. In this study, we explored the feasibility of targeted delivery of an antimitotic drug, monomethyl auristatin E (MMAE), to tumor tissue using a small-molecule based PSMA lig-and. With the aid of Cy5.5, we found that a cleavable linker is vital for the antitumor activity of the ligand–drug conjugate and have developed a new PSMA-targeting prodrug, PSMA-1-VcMMAE. In in vitro studies, PSMA-1-VcMMAE was 48-fold more potent in killing PSMA-positive PC3pip cells than killing PSMA-negative PC3flu cells. In in vivo studies, PSMA-1-VcMMAE significantly inhibited tumor growth leading to prolonged animal survival in different animal models, including metastatic prostate cancer models. Compared to anti-PSMA antibody-MMAE conjugate (PSMA-ADC) and MMAE, PSMA-1-VcMMAE had over a 10-fold improved maximum tolerated dose, resulting in improved therapeutic index. The small molecule–drug conjugates reported here can be easily synthesized and are more cost efficient than anti-body–drug conjugates. The therapeutic profile of the PSMA-1-VcMMAE encourages further clin-ical development for the treatment of advanced prostate cancer.

## 1. Introduction

Prostate cancer is the most common malignancy and the second leading cause of cancer death in men in the United States [1]. Patients with localized disease can be treated with radical prostatectomy and/or radiation therapy [2]. Patients with metastatic prostate cancer can be temporarily treated with androgen deprivation strategies, however, the overwhelming majority of patients with metastatic disease eventually experience disease progression and evolve into a new disease state called metastatic castration-resistant prostate cancer (mCRPC) [3], which has a low survival rate. Since the approval of docetaxel in 2004, eight therapeutics and/or combinations have been approved by the FDA for mCRPC treatment [2,4,5]. However, in randomized controlled trials, generally comparing the treatment group to mitoxantrone, the only FDA approved chemotherapy for advanced prostate cancer before docetaxel’s approval in 2004, survival benefit for all of these agents was less than four months [2]. This year mCRPC is expected to kill 33,330 patients in the United States alone [1], underscoring the need for new therapies.

Cytotoxic drugs are broadly used to treat cancers and have changed the natural course of some cancers [6], however, multidrug resistance and side effects have considerably reduced their usefulness and necessitates the search for more effective chemotherapies. Efforts aimed at improving the quality of treatment for cancer patients have focused on alternative methods to both maintain the effectiveness of chemotherapeutic drugs and minimize systemic toxicity, e.g., targeted drug delivery. Among these novel approaches, the conjugation of cytotoxic agents to humanized antibodies (also known as antibody–drug conjugates, ADCs) has begun to gain momentum among the basic and translational cancer research community [7]. Four ADCs, ado-trastuzumab emtansine (Kadcyla^®^) [8], brentuximab vedotin (Adcetris^®^) [9], inotuzumab ozogamicin (Besponsa) [10], and gemtuzumab ozogamicin (Mylotarg) [11] have been FDA approved and are marketed in the United States; over 30 ADCs are currently undergoing clinical studies [12]. Despite some promising results, there are limitations for ADCs, which have resulted in clinical trial failures. Antibodies themselves can elicit immunogenic responses [13] and ADCs are slowly cleared from nontargeted organs leading to undesired side effects [14,15]. Moreover, the production of antibodies is often low throughput and therefore not cost efficient. Owing to the inherent advantages from their small size, nonimmunogenic nature, and much more manageable synthesis, small organic molecules targeting specific tumor markers have been proposed as a viable alternative to antibodies to synthesize small molecule–drug conjugates (SMDC) for targeted drug delivery [16,17,18].

Prostate-specific membrane antigen (PSMA) is a type II transmembrane protein that is overexpressed (by 100–1000 fold) by virtually all prostate cancers. PSMA expression is further increased in poorly differentiated, metastatic, hormone-refractory carcinomas and in cancer cells from castration-resistant prostate cancer patients [19,20,21,22]. Increased PSMA expression is correlated with the risk of early prostate cancer recurrence after radical prostatectomy [20,23,24]. PSMA is reported to have a robust baseline internalization rate of 60% of its surface PSMA in 2 h, making it an ideal target for imaging and therapy [25]. Two PSMA-targeted ADCs, MLN2704 (PSMA-antibody MLN591-maytansine conjugate) [26] and PSMA-ADC (PSMA-antibody-monomethyl auristatin E (MMAE)) conjugate [27], were reported; however, both of them failed in clinical trials due to toxicity generated during their long circulation in the body. Over the last few years, the number of clinical studies using PSMA ligands to develop radiolabeled imaging agents [28,29,30,31,32,33] and radiotherapeutics, e.g., β-therapy ^177^Lu-PSMA617 [34] and ^177^Lu-PSMA I&T [33]; α-therapy ^225^Ac-PSMA617 [35] and ^213^Bi-PSMA617 [36], has dramatically increased with significant clinical results. These PSMA-targeted agents also demonstrate the superb selectivity of the PSMA ligands for prostate cancers. While many efforts have been focused on the development of PSMA-targeted imaging and radionuclide therapy, there are few examples of small-molecule PSMA-targeted chemotherapeutics [37,38,39,40] and they all used 2-(3-(1,3-dicarboxypropyl)-ureido) pentanedioic acid (DUPA) to deliver the drugs, which has Ki = 8 nM to PSMA receptor [37]. We have previously developed a highly negatively charged PSMA ligand that has binding affinity 5-fold higher than the parent PSMA ligand, (S)-2-(3-((S)-5-amino-1-carboxypentyl)ureido) pentanedioic acid (ZJ24, Ki = 0.3 nM) [37,41], and have achieved excellent results with near-infrared agents, PDT agents, and gold nanoparticles with this negatively charged ligand [42,43,44,45,46,47]. In this study, we have exploited this higher-affinity ligand, PSMA-1 [43], to selectively deliver the very potent microtubule disruption drug, monomethyl auristatin E (MMAE), to prostate cancer cells. The scientific premise of this work is that using a small molecule to selectively target a chemotherapeutic drug to PSMA will improve pharmacokinetics and enable more precise chemotherapy with lower side-effects, thusly eliminating the hurdles that prevented PSMA-ADC from moving forward.

Having a ligand with high binding affinity to PSMA, the other two major components for ADC and SMDC design are payloads and linker. For payload selection, the best approaches are those with extremely potent drugs that have a low-nanomolar or subnanomolar EC_50_ [18]. The most commonly used drugs include MMAE [9,48,49] and maytansine [8,50,51]. We selected MMAE as our payload because of its high potency and demonstrated efficacy in ADCs including brentuximab vedotin for different types of cancers [9,48,49]. The other vital component is the linker that forms a chemical connection between the ligand and the drug. An ideal linker should be sufficiently stable in the circulation to allow the drug to remain attached to the binding moiety, but at the same time should allow efficient active drug once the conjugate is taken up by the cancer cells [52,53]. Conventionally, there are two families of linkers, cleavable and noncleavable linkers. For cleavable linkers, the goal is that the chemical bond formed between the binding moiety and the payload is cleaved intracellularly releasing intact active drug. The most commonly used release mechanisms for linkers are protease-sensitivity, pH-sensitivity, and glutathione-sensitivity. If employed correctly the use of cleavable linkers can result in a targeted drug that is inactive until the drug is released, i.e., a prodrug. For noncleavable linkers, the conjugates enter the cells and there is no release mechanism, i.e., the conjugate has full drug activity [52,54]. In this study we sought to develop a prodrug approach, first determining the impact of a cleavable self-immolative maleimido-caproyl-Val-Cit-PABC linker (Vc) and noncleavable maleimido-caproyl linker (Mc) on antitumor activity of ligand–drug conjugates with the aid of a near-infrared (NIR) fluorescent dye Cy5.5 labeling. Our results showed that the drug release is possible and crucial for the antitumor activity. Further in vivo antitumor activity studies in heterotopic, orthotopic, and metastatic prostate cancer models in mice showed that PSMA-targeted prodrug strategy selectively and effectively inhibited PSMA-expressing tumor growth and prolonged animal survival with no obvious toxicity. More importantly, PSMA-1-VcMMAE also had a much more favorable therapeutic index than either MMAE or PSMA-ADC.

## 2. Results

### 2.1. A Prodrug Strategy Is Crucial for Antitumor Activity

#### 2.1.1. Binding Affinity of PSMA-Targeted MMAE-Cy5.5 Conjugates

**Figure 1:**
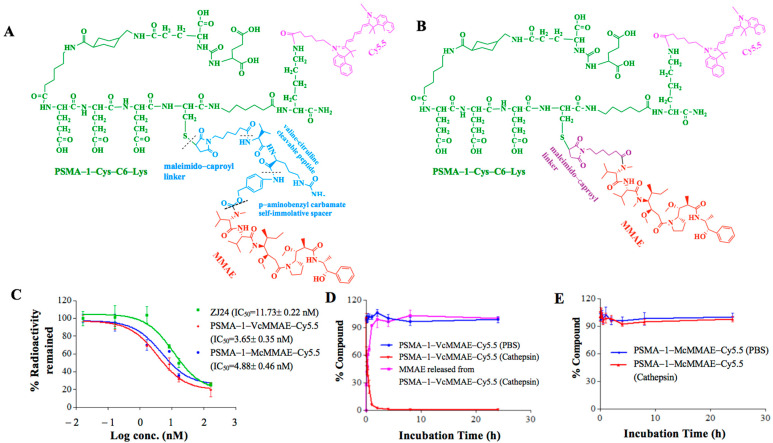
Novel Prostate specific membrane antigen (PSMA)-targeted drug conjugates. (**A**) Structure of PSMA-1-VcMMAE-Cy5.5 which has a cleavable linker (in blue color). (**B**) Structure of PSMA-1-McMMAE-Cy5.5 which has a noncleavable linker (in purple color). (**C**) In vitro competition binding results of PSMA-targeted drug conjugates. Values are mean ± SD of triplicates. PSMA-1-VcMMAE-Cy5.5 and PSMA-1-McMMAE-Cy5.5 showed similar binding affinity. (**D**) In vitro cathepsin cleavage of PSMA-1-VcMMAE-Cy5.5. Values are mean ± SD of triplicates. (**E**) In vitro cathepsin cleavage of PSMA-1-McMMAE-Cy5.5. Values are mean ± SD of triplicates.

To determine which linker would be most suitable, we synthesized PSMA-1-VcMMAE-Cy5.5 with a cathepsin cleavable linker (Figure 1A) and PSMA-1-McMMAE-Cy5.5 with noncleavable linker (Figure 1B). In both molecules Cy5.5 was linked to the terminal Lys residue on PSMA-1-Cys-C6-Lys to allow visualization of the drug conjugates. Our PSMA-1 ligand is rationally designed based on the fact that the S1 binding pocket of PSMA is arginine-rich and is highly positively charged [43]. The ligand has three D-glutamic acids in the structure to form strong ion pairs with the positively charged guanidine groups of arginine in the substrate binding pocket of PSMA. A C6-linker is included to optimize the steric hindrance, thus leading to better fit and binding characteristic [43]. Competition binding experiments demonstrated that the complexity of the drug conjugates did not impact their binding affinity to PSMA; PSMA-1-VcMMAE-Cy5.5 showed an IC_50_ of 3.65 nM and PSMA-1-McMMAE-Cy5.5 had an IC_50_ of 4.88 nM, both similar to unconjugated PSMA-1, IC_50_ = 2.30 nM [43] and significantly lower than the parent ZJ24 ligand [41], IC_50_ = 11.73 nM (Figure 1C). These results concurred with our previous studies that inclusion of a large group to PSMA-1 ligand does not impact its binding affinity to PSMA [43,44].

#### 2.1.2. Cathepsin Cleavage of PSMA-Targeted MMAE-Cy5.5 Conjugates

Cathepsin is a prevalent protease that PSMA internalized ligands will encounter as they enter into late endosomes/lysosomes [55]. To investigate if PSMA-targeted drug conjugates were cleavable by cathepsin B, the two conjugates were incubated with or without cathepsin protease and chromatographed by high performance liquid chromatography (HPLC). When the conjugates were incubated with PBS, both PSMA-1-VcMMAE-Cy5.5 (Figure 1D) and PSMA-1-McMMAE-Cy5.5 (Figure 1E) were stable. In the presence of cathepsin, PSMA-1-VcMMAE-Cy5.5 degraded rapidly with a half-life at 0.33 h, releasing intact MMAE, while PSMA-1-McMMAE-Cy5.5 remained intact (Figure 1D,E and Figure S1). The results demonstrated that PSMA-1-VcMMAE-Cy5.5 can be cleaved by cathepsin, while PSMA-1-McMMAE-Cy5.5 is not cleavable by the protease.

#### 2.1.3. Cellular Uptake Studies

**Figure 2:**
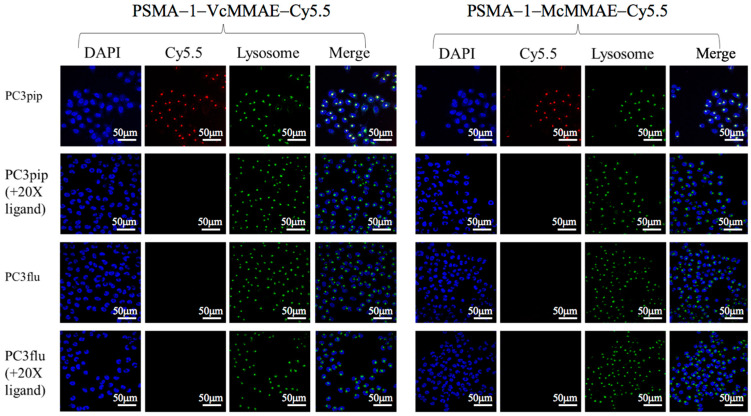
In vitro fluorescence studies of PSMA-1-MMAE-Cy5.5 conjugates. In vitro cellular uptake results of PSMA-targeted drug conjugates. Cells were incubated with 50 nM of drug conjugates for 4 h. Nuclei were stained by DAPI and are false colored blue, lysosomes were detected by LysoOrange and are false colored green, and drug conjugates are false colored red. Selective uptake was observed only in PC3pip cells and the conjugates were mainly located in lysosomes. Specificity of drug conjugates for PSMA binding was evaluated by including 1 mM of unlabeled PSMA-1 ligand during incubations. Signal in PC3pip cells was significantly competed by PSMA-1, suggesting the binding is selective. Images are taken at 40×. Representative images are shown from three independent experiments.

To determine if PSMA-targeted drug conjugates would result in selective cellular binding and uptake, in vitro uptake studies were performed in both PSMA-positive PC3pip and PSMA-negative PC3flu cells. Similar levels of fluorescence uptake into PC3pip cells were observed after treatment with either PSMA-1-VcMMAE-Cy5.5 or PSMA-1-McMMAE-Cy5.5 and the fluorescence signals were localized to the lysosomal compartment of the cells, which was detected by LysoOrange (Figure 2 and Figure S2), while no fluorescence signal was observed in PC3flu cells. Presence of an excess amount of PSMA-1 ligand completely blocked the fluorescence in PC3pip cells, indicated that binding of the two conjugates was selective for the PSMA receptor expressed on the PC3pip cells.

#### 2.1.4. Disruption of α-Tubulin

**Figure 3:**
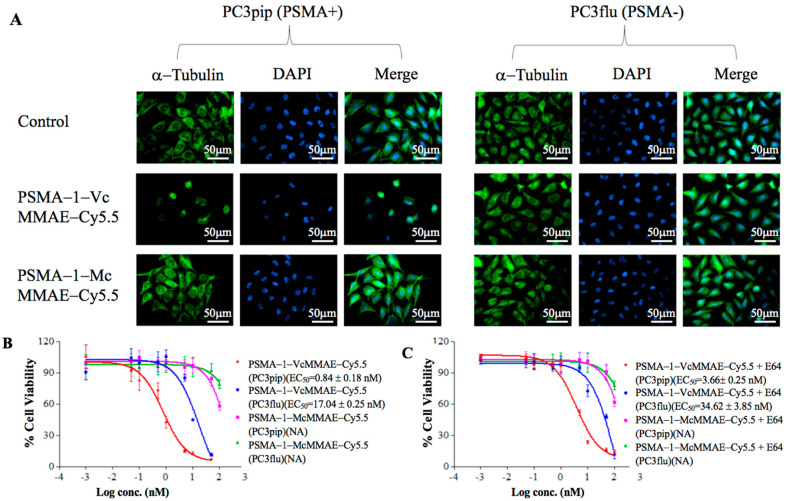
In vitro disruption of α-tubulin and cytotoxicities of PSMA-1-MMAE-Cy5.5 conjugates. (**A**) Immuno-detection of α-tubulin. Cells were treated with 5 nM of drugs for 24 h then fixed and stained by Alexa Fluor 488-labeled α-tubulin antibody (false color green). Selective disruption in PC3pip cells was observed by immunofluorescence only when cells were treated with PSMA-1-VcMMAE-Cy5.5. Images were taken at 40X. Representative images are shown from three independent experiments. (**B**) In vitro cytotoxicity of PSMA-1-VcMMAE-Cy5.5 and PSMA-1-McMMAE-Cy5.5 to PSMA-positive PC3pip cells and PSMA-negative PC3flu cells after 72-h incubation. Values are mean ± SD of six replicates. (**C**) In vitro cytotoxicity of PSMA-1-VcMMAE-Cy5.5 and PSMA-1-McMMAE-Cy5.5 to PC3pip and PC3flu cells after 72-h incubation in the presence of E64, a protease inhibitor. Values are mean ± SD of six replicates.

It is known that MMAE inhibits cell division by destabilization of α-tubulin [56]. To further validate the biological consequence of MMAE-conjugate treatment, in vitro immunofluorescence staining of α-tubulin was performed (Figure 3A). Incubation with PSMA-1-VcMMAE-Cy5.5 led to selective tubulin disruption of the microtubule network in PC3pip, causing them to round up and lose the spindle structure; conversely, no obvious changes in α-tubulin structure was observed in PC3flu cells treated with PSMA-1-VcMMAE-Cy5.5. In contrast to PSMA-1-VcMMAE-Cy5.5, no apparent disruption of tubulin was observed in PC3pip or PC3flu cells when treated with the same concentration of the noncleavable PSMA-1-McMMAE-Cy5.5, suggesting drug release is required for activity.

#### 2.1.5. In Vitro Cytotoxicity of PSMA-Targeted MMAE-Cy5.5 Conjugates

To compare the potency of PSMA-1-VcMMAE-Cy5.5 and PSMA-1-McMMAE-Cy5.5, cytotoxicity studies were performed in both PSMA-positive PC3pip and PSMA-negative PC3flu cells. PSMA-1-VcMMAE-Cy5.5 was about 20-fold more potent at killing PSMA-positive PC3pip cells (EC_50_ = 0.84 nM) than PSMA-negative PC3flu cells (EC_50_ = 17.0 nM) (Figure 3B). In contrast, PSMA-1-McMMAE-Cy5.5 was ineffective for cell killing and no EC_50_ values could be obtained for either PC3pip or PC3flu cells for the concentrations tested. These results agreed with the cellular uptake and disruption of α-tubulin studies. To confirm protease dependent activation of PSMA-1-VcMMAE-Cy5.5, we performed in vitro cell killing studies including 50 μM of a wide spectrum protease inhibitor E64 [57] (Figure 3C). Coincubation with 50 μM of protease inhibitor E64 reduced the potency of PSMA-1-VcMMAE-Cy5.5 to kill PC3pip cells from 0.84 to 3.66 nM, suggesting PSMA-1-VcMMAE-Cy5.5 acts as a prodrug dependent on protease activation.

#### 2.1.6. In Vivo Fluorescence Imaging of PSMA-Targeted MMAE-Cy5.5 Conjugates

**Figure 4:**
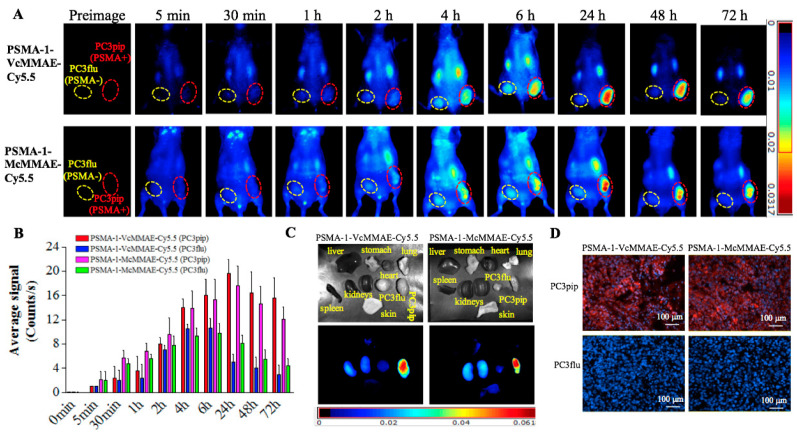
In vivo fluorescence image of PSMA-1-MMAE-Cy5.5. (**A**) In vivo Maestro imaging of a typical mouse bearing heterotopic PC3pip and PC3flu tumors treated with 40 nmol/kg of drug conjugates through *i.v.* injection. Representative images are shown of *n* = 5. Selective uptake was observed in PC3pip tumors. (**B**) Quantification of fluorescent signal intensity in PC3pip and PC3flu tumors. Values are mean ± SD of five animals. (**C**) Ex vivo imaging of mouse organs at 72-h post-injection. Fluorescent signal in PC3pip tumor was significantly higher than in other organs. Representative images are shown of *n* = 5. (**D**) Representative fluorescence images of sectioned tumors. DAPI is false colored blue and Cy5.5 is false colored red. Cy5.5 fluorescence signal was observed in PC3pip tumors (*n* = 5).

To demonstrate selective tumor uptake in vivo, mice bearing both PC3flu and PC3pip tumors were injected with 40 nmol/kg of PSMA-1-VcMMAE-Cy5.5 or PSMA-1-McMMAE-Cy5.5 and uptake was monitored via fluorescence imaging over time (Figure 4). Similar selective uptake was observed in PC3pip tumors for both conjugates, peaking at 24-h post-injection followed by a prolonged clearance. At 24-h post-injection there was little to no detectable uptake in PSMA-negative PC3flu tumors (Figure 4A,B). Ex vivo imaging of tissues at 72-h post-injection showed that fluorescence was mainly retained in the PSMA-expressing tumor; no/minimal fluorescence was detected in other organs (Figure 4C). Further fluorescence imaging of sectioned tumors showed that fluorescence signal was only observed in PC3pip tumor (Figure 4D).

#### 2.1.7. In Vivo Antitumor Activity of PSMA-Targeted MMAE-Cy5.5 Conjugates

**Figure 5:**
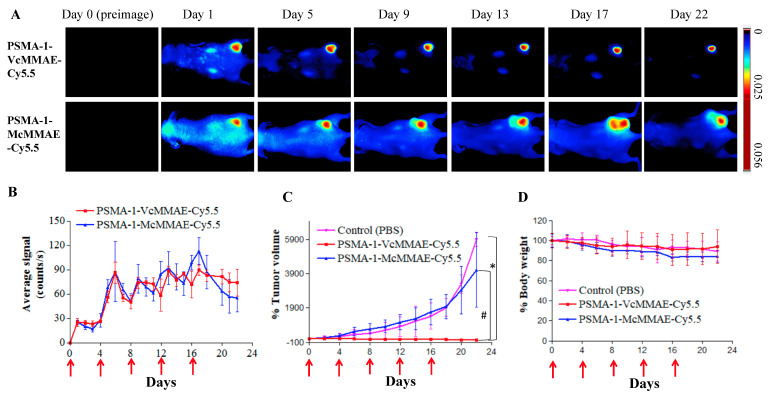
Treatment of mice bearing heterotopic PC3pip tumor with 160 nmol/kg of PSMA-1-MMAE-Cy5.5 conjugates. Mice received 160 nmol/kg of either PSMA-1-VcMMAE-Cy5.5 or PSMA-1-McMMAE-Cy5.5 every 4 days with a total of five doses (as indicated by the red arrows). (**A**) Fluorescence images of mice bearing PC3pip tumor treated with PSMA targeted drug conjugates on different days. Bright fluorescent signal was observed on PC3pip tumors. Representative images are shown of *n* = 5. (**B**) Quantification of average fluorescent signal on PC3pip tumors. The average fluorescent signals on the tumors were about the same for both conjugates. Values are mean ± SD, *n* = 5. (**C**) In vivo tumor inhibition of PSMA-targeted drug conjugates using heterotopic PC3pip tumors. Significant tumor regression was only observed in mice treated with PSMA-1-VcMMAE-Cy5.5. Values represent mean ± SD of five animals. (*, PSMA-1-VcMMAE-Cy5.5 vs. PBS, *p* < 0.05; #, PSMA-1-VcMMAE-Cy5.5 vs. PSMA-1-McMMAE-Cy5.5, *p* < 0.05). (**D**) Body weight changes of mice treated with PSMA-1-drug conjugates. No significant body weight loss was observed between the three groups. Values are mean ± SD, *n* = 5.

**Figure 6:**
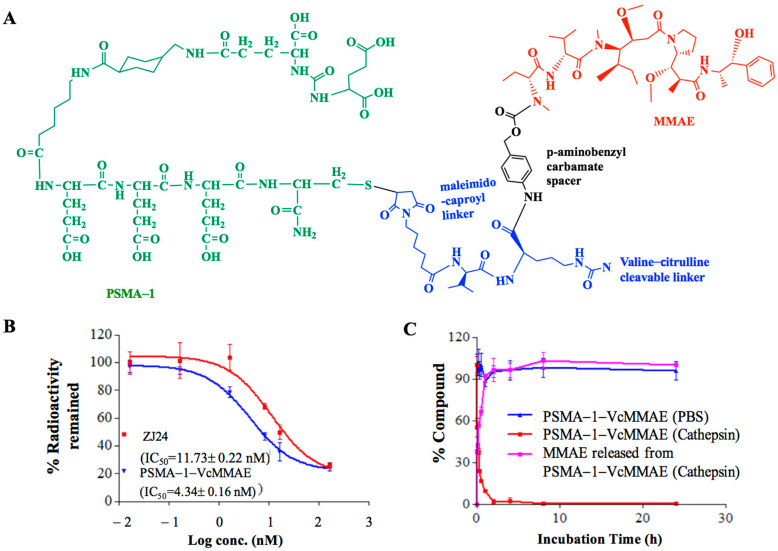
In vitro characterization of PSMA-1-VcMMAE. (**A**) Structure of PSMA-1-VcMMAE. (**B**) In vitro competition binding results of PSMA-1-VcMMAE. Values are mean ± SD of triplicates. (**C**) In vitro cathepsin cleavage of PSMA-1-VcMMAE. Values are mean ± SD of triplicates.

We next compared the antitumor activity of the two conjugates in mice bearing a flank PC3pip tumor. Each animal received 160 nmol/kg of PSMA-1-VcMMAE-Cy5.5 or PSMA-1-McMMAE-Cy5.5 through tail vein injection every 4 days with a total of five doses. (Dosing was based on previously published dose and schedules for antibody–drug conjugates [27,49].) Mice were then imaged, and tumors were measured by caliper. Quantitative Maestro fluorescence imaging showed that the average fluorescence signal in PC3pip tumors was similar for PSMA-1-VcMMAE-Cy5.5 and PSMA-1-McMMAE-Cy5.5 and peaked in PC3pip tumors 24-h after each injection (day 1, day 5, day 9, day 13, and day 17) (Figure 5A,B). The fluorescence from Cy5.5 also reflected the tumor size change during the treatment (Figure 5A). Untreated PC3pip tumor grew rapidly achieving a 50-fold increase in size by day 22 (Figure 5C). Mice treated with the noncleavable conjugate, PSMA-1-McMMAE-Cy5.5, showed similar growth rate as that measured for untreated mice. In contrast, administration of PSMA-1-VcMMAE-Cy5.5 effectively inhibited tumor growth as early as day 6 after the initial dose. Caspase 3 staining of tumors extracted on day 4 of treatment also showed that PSMA-1-VcMMAE-Cy5.5 induced significantly more apoptosis than PSMA-1-McMMAE-Cy5.5 (Figure S3). These results suggested that prodrug strategy with cleavable linker is imperative for the antitumor activity of the conjugates. The changes in body weight were similar between mice receiving PBS or targeted drug conjugates, suggesting that the drug treatment is not overtly toxic to the animals (Figure 5D). Further, in vivo antitumor activity studies of PSMA-1-VcMMAE-Cy5.5 showed that it effectively inhibited PC3pip tumor growth and prolonged animal survival in a dose dependent manner without loss of body weight (Figure S4A–C)). In the group treated with 160 nmol/kg of PSMA-1-VcMMAE-Cy5.5, three out of five mice were tumor-free at end of the 90-day study and two mice had tumors grow back. It was found that treatment of mice that had extremely large tumors (≈2000 mm^3^) with PSMA-1-VcMMAE-Cy5.5 was able to ablate the tumor (Figure S4D). These data demonstrated the efficacy of PSMA-1-VcMMAE-Cy5.5 with the prodrug strategy. Cy5.5 is helpful in preclinical studies for demonstrating and understanding the mechanism or the prodrug, however, its application for clinical translation is less obvious and will add cost to drug synthesis. We therefore designed and tested a prodrug molecule without Cy5.5 named PSMA-1-VcMMAE (Figure 6A).

### 2.2. Efficacy of PSMA-1-VcMMAE

#### 2.2.1. In Vitro Characterization of PSMA-1-VcMMAE

In vitro competition binding studies showed that PSMA-1-VcMMAE had an IC50 at 4.34 nM, which is similar to PSMA-1-VcMMAE-Cy5.5 (Figure 6B). PSMA-1-VcMMAE was stable in PBS and incubation with cathepsin resulted in release of free MMAE from the conjugate (Figure 6C).

In in vitro cytotoxicity studies, PSMA-1-VcMMAE selectively killed PC3pip cells with EC_50_ at 4.64 nM and it was 48-fold more potent for PC3pip cells than for PC3flu cells (Table 1). In contrast, free MMAE showed no selectivity between PC3pip and PC3flu cells. In the presence of 10 μM of PSMA-1, the potency of PSMA-1-VcMMAE to kill PC3pip cells was reduced, indicating that the killing is dependent on PSMA binding. PSMA-1-VcMMAE activity was also susceptible to E64 protease inhibition suggesting that it worked as a prodrug (Table 1). We compared the cytotoxicity of PSMA-1-VcMMAE with antibody-conjugated MMAE (PSMA-ADC), which entered clinical trials but failed due to toxicity [27,58]. It was found that PSMA-ADC was more potent for PC3pip cells (EC_50_ = 0.063 nM) and had better selectivity defined by differential cytotoxicity ratio between PC3pip and PC3flu cells of 3838-fold compared to PSMA-1-VcMMAE (48-fold). PSMA-ADC (EC_50_ = 241.8 nM) and PSMA-1-VcMMAE (EC_50_ = 221.7 nM) had similar potency to kill non-PSMA expressing PC3flu cells, suggesting the antibody’s greater affinity resulted in efficacy differences against PC3pip cells. Further cytotoxicity studies were performed in other cell lines that express different levels of PSMA (Figure S5); our results showed that cytotoxicity of PSMA-1-VcMMAE was correlated with PSMA expression level. The same results were observed with PSMA-1-VcMMAE-Cy5.5.

#### 2.2.2. Maximum Tolerated Dose of PSMA-1-VcMMAE

**Figure 7:**
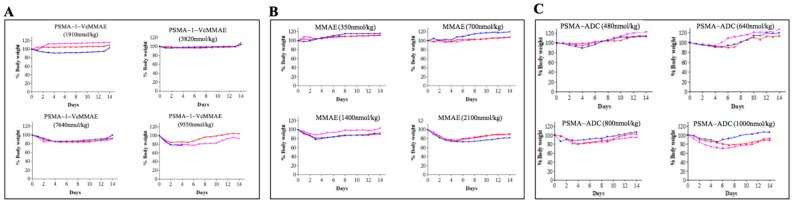
Determination of maximum tolerated dose (MTD) of drugs. Tumor free male nude mice received a single dose of PSMA-1-VcMMAE (**A**), MMAE (**B**), and PSMA-ADC (**C**). Loss of 20% of body weight was used as the criteria to determine MTD. Each dose group had three mice as indicated in red, blue, and pink colors.

The maximum tolerated dose (MTD) was determined in tumor-free athymic nude mice to determine the toxicity of the conjugates. Loss of 20% body weight or any overt signs of toxicity was used as an end point. We compared the MTD of free drug MMAE, PSMA-1-VcMMAE, and PSMA-ADC. MTDs of single dose IV. injection of MMAE, PSMA-1-VcMMAE, and PSMA-ADC were 700, 7640, and 640 nmol/kg, respectively (Figure 7), highlighting the superior safety (10-fold or greater) of PSMA-1-VcMMAE compared to all the other drug derivatives.

#### 2.2.3. In Vivo Antitumor Activity of PSMA-1-VcMMAE

**Figure 8:**
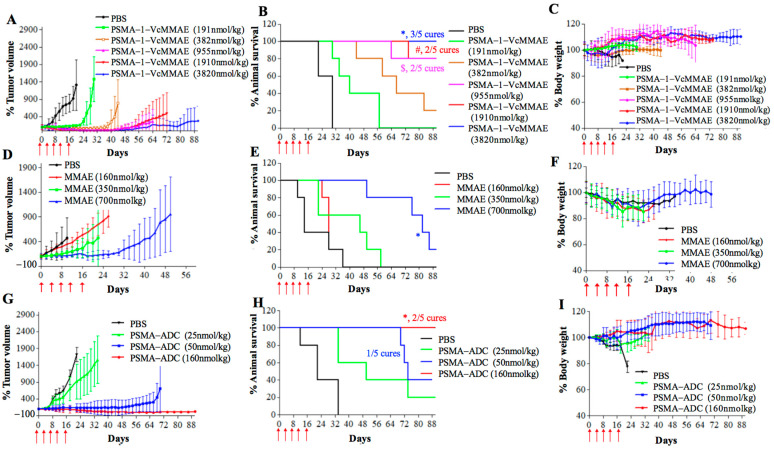
Dose response of antitumor activity in mice bearing heterotopic PC3pip tumors. Mice received drug through tail vein injection. Treatment were scheduled every 4 days with a total of five doses as indicated by the red arrow. Each group had five mice. For tumor growth curves and body weight curves, values are mean ± SD of five animals. The plots stopped when animals died during the experiments since values are represent as mean ± SD of five animals. (**A**) Tumor growth curves of mice treated with PSMA-1-VcMMAE. (**B**) Kaplan–Meier survival curves of mice treated with PSMA-1-VcMMAE. ($, 955 nmol/kg vs. PBS, *p* = 0.0046; #, 1910 nmol/kg vs. PBS, *p* = 0.0004; *, 3820 nmol/kg vs. PBS, *p* = 0.0004). (**C**) Body weight changes of mice treated with PSMA-1-VcMMAE. (**D**) Tumor growth curves of mice treated with MMAE. (**E**) Kaplan–Meier survival curves of mice treated with MMAE. (*, 700 nmol/kg vs. PBS, *p* = 0.0005). (**F**) Body weight changes of mice treated with PSMA-1-VcMMAE. (**G**) Tumor growth curves of mice treated with PSMA-ADC. (**H**) Kaplan–Meier survival curves of mice treated with PSMA-ADC. (*, 160 nmol/kg vs. PBS, *p* = 0.0002). (**I**) Body weight changes of mice treated with PSMA-ADC.

For in vivo antitumor activity, we first studied in vivo potency of PSMA-1-VcMMAE in nude mice bearing heterotopic PC3pip tumors. Mice received drugs intravenously every 4 days with a total of five doses. In PBS control groups the tumor grew rapidly resulting in animal death within 30 days (Figure 8). In contrast, treatment with PSMA-1-VcMMAE showed the ability to inhibit tumor growth and prolong animal survival in a dose dependent manner. At the lowest dose of 191 nmol/kg, significant tumor inhibition was observed and at the highest dose tested (3820 nmol/kg, ½ of its MDT), all five mice survived the 90-day experimental time and three out of five mice were tumor free, resulting in 60% cure. No body weight loss was observed even at the highest dose tested (Figure 8C). Furthermore, no noticeable histological changes were observed in Hematoxylin and Eosin (H&E) staining of major organs in mice treated with 3820 nmol/kg of PSMA-1-VcMMAE on day 21 with only microscopic tubular degeneration/atrophy observed in testis, indicating that PSMA-1-VcMMAE treatment is well-tolerated (Figure S6). Compared to PSMA-1-VcMMAE, MMAE did not show any antitumor activity at the dose of 160 nmol/kg, efficacy of MMAE was only observed at its MTD 700 nmol/kg (Figure 8D–F). PSMA-ADC showed the ability to effectively inhibit tumor growth and extend animal survival at the dose of 50 nmol/kg (Figure 8G–I). Doses for PSMA-1-VcMMAE of 382 nmol/kg, MMAE of 700 nmol/kg, and PSMA-ADC of 50 nmol/kg were the lowest effective drug concentrations to inhibit tumor growth for 30 days; therefore, the therapeutic indexes, which were defined as the ratio of the minimal effective dose that can inhibit tumor growth to its maximum tolerated dose, of PSMA-1-VcMMAE, MMAE, and PSMA-ADC were 20, 1, and 12.8, respectively (Figure S7, Table S1). Targeting PSMA using small molecular PSMA ligand, therefore, improved the therapeutic index as compared to MMAE and PSMA-ADC. It was also found that PSMA-1-VcMMAE was significantly more effective at inhibiting PSMA-positive PC3pip tumor growth than PSMA-negative PC3flu tumor growth when used at the dose of 955 nmol/kg (*p* = 0.0493) (Figure S8), indicating selective killing of PSMA-1-VcMMAE by targeting PSMA.

To show versatility of the antitumor activity of PSMA-1-VcMMAE, we also tested it in mice bearing heterotopic C4-2 tumors, which are androgen-independent prostate cancer cells endogenously expressing PSMA. At the dose of 1910 nmol/kg, PSMA-1-VcMMAE showed the ability to successfully inhibit C4-2 tumor growth with no weight loss and prolonged animal survival significantly (*p* = 0.0018) (Figure S9) with a 40% cure rate.

**Figure 9:**
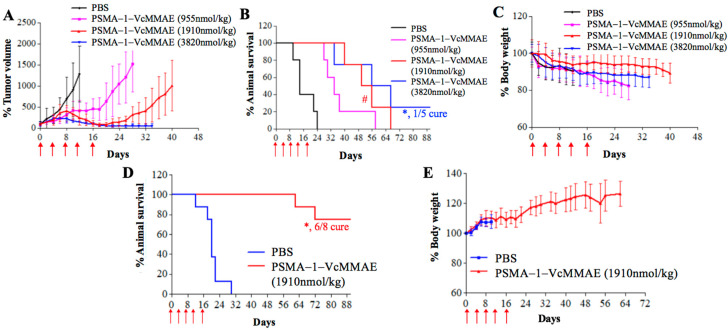
In vitro antitumor activity studies of PSMA-1-VcMMAE in orthotopic (**A**–**C**) (*n* = 5) and metastatic tumor models (**D**,**E**) (*n* = 8). Mice received drug through tail vein injection. Treatments were scheduled every 4 days with a total of five doses as indicated by the red arrow. (**A**) Orthotopic PC3pip tumor growth curves of mice treated with PSMA-1-VcMMAE. Each group had five mice. Values are mean ± SD of five animals. The plots stopped when animals died during the experiments since values are represent as mean ± SD of five animals. (**B**) Kaplan–Meier survival curves of mice bearing orthotopic PC3pip tumor. (#, 1910 nmol/kg vs. PBS, *p* = 0.0449; *, 3820 nmol/kg vs. PBS, *p* = 0.0019). (**C**) Body weight changes of mice treated with PSMA-1-VcMMAE. Values are mean ± SD of five animals. The plots stopped when animals died during the experiments since values are represent as mean ± SD of five animals. (**D**) Kaplan–Meier survival curves of mice bearing metastatic PC3pip tumor. (*, 1910 nmol/kg vs. PBS, *p* = 0.0033). (**E**) Body weight changes of mice bearing metastatic PC3pip tumor treated with PSMA-1-VcMMAE. Values are mean ± SD of eight animals. The plots stopped when animals died during the experiments since values are represent as mean ± SD of eight animals.

We next investigated the efficacy of PSMA-1-VcMMAE in mice bearing orthotopic PC3pip tumors, which mimic human prostate cancer in a more realistic way. Inhibition of tumor growth (Figure 9A) and extension of animal survival time were observed in the orthotopic PC3pip tumor models (Figure 9B), and significant differences were observed at the dose of 1910 (*p* = 0.0449) and 3860 nmol/kg (*p* = 0.0019) when compared to the PBS control, with one mouse that was tumor free at the dose of 3860 nmol/kg. Treatment with PSMA-1-VcMMAE did not cause significant body weight changes (Figure 9C).

The mortality of prostate cancer is mainly due to metastasis and metastatic castration resistant prostate cancer is the most deadly and difficult form of the disease to treat. To test the effectiveness of PSMA-1-VcMMAE against metastatic disease, we developed a metastatic prostate cancer model using intracardiac injection of GFP-expressing PC3pip cells, which are castration resistant. As Figure S10 shows, this model results in significant metastatic disease 4 weeks after cardiac injection. We then used this model to assess the effectiveness of our drug conjugate. Treatment was initiated 1 week after cardiac injection of tumors cells, and mice received 1910 nmol/kg of PSMA-1-VcMMAE every 4 days with a total of five doses. Control mice died within 30 days. In contrast, treatment of PSMA-1-VcMMAE significantly extended animal survival resulting in 75% cure rate (six out of eight, *p* = 0.0003 as compared to control mice) with no body weight loss during the 90-day experimental period (Figure 9D,E).

## 3. Discussion

During early development of ADCs, protease cleavable linkers were predicted to confer exceptional control over payload release upon exposure to proteases [59]. The Vc linker is one of the most widely used linkers in ADC design, being included in the majority of MMAE-based ADCs in clinical studies [60]. The Vc linker has also been used in SMDC development [61,62]. However, consistent toxicity has been reported in ADCs utilizing Vc-MMAE, which is related to instability of Vc linker in plasma and long blood half-life of ADCs [63]. Conversely, the greatest advantage of the noncleavable linkers is their increased plasma stability [63]. Recently, antitumor activities of MMAE based ADCs with noncleavable linker have been reported [64,65] and it was found that complete lysosomal proteolytic degradation of the antibody is required to generate toxic payloads for ADCs with noncleavable linker. In this study, we used our PSMA-1 ligand to deliver MMAE to PSMA-expressing prostate cancer cells. To determine which linker would be most suitable, we synthesized both the cathepsin cleavable PSMA-1-VcMMAE-Cy5.5 conjugate (Figure 1A) and the noncleavable PSMA-1-McMMAE-Cy5.5 conjugate (Figure 1B). The fluorescence of Cy5.5 allowed straightforward comparison of our conjugates in vitro and in vivo, providing detailed understanding of importance of the linker. The results from the in vitro cellular uptake assay showed that when cells were treated with either PSMA-1-VcMMAE-Cy5.5 or PSMA-1-McMMAE-Cy5.5, fluorescent signal was only observed in PSMA-positive PC3pip cells but not in PSMA-negative PC3flu cells (Figure 2). The signal in PC3pip cells was blocked in the presence of excess amount of PSMA-1 ligand. These results indicated that both conjugates bound selectively to PSMA. In vivo fluorescence imaging also showed that both conjugates were selectively accumulated in PSMA-positive PC3pip tumor cells with similar biodistribution profiles (Figure 4). Although both conjugates selectively accumulated in PC3pip cells in vitro and in vivo, effective antitumor activity was only observed when the cleavable linker was employed in drug design (Figure 5). Release of free MMAE was observed when PSMA-1-VcMMAE-Cy5.5 was incubated with cathepsin, while at the same condition, PSMA-1-McMMAE-Cy5.5 remained intact (Figure 1D,E and Figure S1). In addition, the presence of protease inhibitor E64 reduced the cytotoxicity of PSMA-1-VcMMAE-Cy5.5 to PC3pip more than 4.3-fold, while E64 had no effect on the cytotoxicity of PSMA-1-McMMAE-Cy5.5 (Figure 3B,C). Our results indicated that cleavable linker that can release free drug effectively and is crucial to the antitumor activity of our PSMA ligand–drug conjugate. Once entering the cells, the conjugates located mainly in the lysosomes (Figure 2), where cathepins are highly expressed and active. The protease will liberate free MMAE resulting in disruption of α-tubulin and cancer cell death (Figure 3A). Recently Lv et al. reported PSMA targeting paclitaxel conjugates using PSMA ligand DUPA [38]. They reported that the PTX-SS-DUPA conjugate employing a disulfide cleavable linker was more potent than PTX-DUPA with a noncleavable linker, which concurs with our results. One possible explanation that cleavable linker is crucial for SMDC design is that SMDC cannot undergo complete proteolytic degradation to exert antitumor activity. The reported mechanism of action of MMAE is simultaneous binding to tubulin to each end of MMAE [66]. Together these data suggest that the conjugated ligand impedes binding until fully removed.

Having confirmed that the cleavable linker is essential for PSMA-targeted SMDC, we then studied the antitumor activity of PSMA-1-VcMMAE. The payload and linker in PSMA-1-VcMMAE resembles the structure of a PSMA antibody-MMAE conjugate (PSMA-ADC) which was withdrawn from clinical trial due to toxicity [27,58]. Here, by replacing the antibody with a PSMA targeting ligand (PSMA-1) we developed a small-molecule–drug conjugate to reduce the cost and shorten the circulation time, potentially reducing the off-target toxicities resulting from longer blood half-lives. In vitro comparison of the cytotoxicity of free MMAE with PSMA-1-VcMMAE and PSMA-ADC (Table 1) showed that free MMAE killed both PC3pip and PC3flu cells with no selectivity. In contrast, PSMA-ADC and PSMA-1-VcMMAE selectively killed PC3pip cells with PSMA-ADC being the most potent and selective. The improved potency and selectivity of PSMA-ADC may be due to its significantly higher affinity for PSMA (picomolar) [27] than the PSMA-1 ligand used (nanomolar).

The maximum tolerated doses (MTD) of MMAE, PSMA-ADC, and PSMA-1-VcMMAE using 20% body weight loss as the reference were 700, 7640, and 640 nmol/kg, respectively (Figure 7). Interestingly, the MTD for PSMA-ADC was similar to MMAE and was much lower than our nanomolar affinity small molecule conjugate PSMA-1-VcMMAE. Further antitumor activity studies using mice bearing heterotopic PC3pip tumors showed that PSMA-1-VcMMAE effectively inhibited tumor growth at the dose of 382 nmol/kg compared to MMAE at 700 nmol/kg and PSMA-ADC at 50 nmol/kg (Figure S7). Therefore, PSMA-1-VcMMAE had a superior therapeutic index compared to MMAE and PSMA-ADC (Table S1). It was noticed that animals treated by PSMA-1-VcMMAE showed no body weight loss and no histological damage to major organs including salivary gland and kidney, indicating low toxicity of the treatment. It is speculated that replacement of antibody by small molecule ligand changes the pharmacokinetics of the conjugates, resulting in overall improved therapeutic index of PSMA-1-VcMMAE.

Notably, PSMA-1-VcMMAE showed the ability to significantly prolong the survival time of animals not only in heterotopic and orthotopic PC3pip tumors, but also in the metastatic PC3pip tumor model. Treatment was initiated one week after tumor inoculation, before the metastasis was detectable using imaging, and successfully treated tumor metastasis. As a significant percentage of patients with prostate cancer die from the metastatic form of the disease due to lack of effective treatment options, it is critical to develop potent treatments that can effectively eradicate cancer cells and control metastatic tumors. The work reported here may open an intriguing and promising avenue of therapy for patients, with applications across the natural history of prostate cancer. 

Currently, there are very few PSMA-targeted chemotherapeutic agents reported, four of them entered clinical trials including antibody-based MLN2704 (PSMA-antibody MLN591-DM1 conjugate) [26] and PSMA-ADC [27], small ligand based EC1169 [37,40], and nanoparticle based BIND014 [67], but all failed. MLN2704 and PSMA-ADC released free drug during their long circulation in the body and caused unwanted side effects. EC1169 used a reducible disulfide linker to conjugate PSMA ligand DUPA to tubulysin B, although active in animals, it showed lack of activity in patients. BIND014 increased intratumoral docetaxel and inhibited tumor growth in animals, but its clinical activity and toxicity did not appear to differ substantially from that of free docetaxel. Recently, DUPA-indenoisoquinoline [39] and DUPA–paclitaxel conjugates [38] were reported. While their data indicated effective targeting, they did not demonstrate increased efficacy of the targeted agent, the targeted drug only approached the effective concentrations of unconjugated paclitaxel. Compared to these reports, PSMA-1 (binding affinity is 4–5-fold better than ZJ24 whose Ki = 0.3 nM) [41] has better binding affinity than DUPA (Ki = 8.0 nM) [37], which will improve the overall targeting efficacy. PSMA-1-VcMMAE utilizes the same targeting strategy as PSMA-ADC, but has shorter circulation time due to its smaller size and can be easily and more rapidly excreted from the body [43,44] leading to less off target drug activation and reduced toxicity. By replacing the antibody with our PSMA-1 ligand, PSMA-1-VcMMAE dramatically improved the therapeutic index. These characteristics will help it avoid the problems of PSMA-ADC found in clinical trials.

Compared to very few PSMA-targeted chemotherapeutics, many PSMA-targeting radionuclides have been developed and entered into clinical trials for the treatment of metastatic prostate cancer [34,35,36,68,69,70]. Although promising results have been achieved, one of the major concerns is xerostomia due to nonspecific uptake of the radionuclide in the salivary gland leading to damage of the gland [71], which severely impairs the quality of life of patients. In contrast to PSMA-based radionuclide therapy, no histological damage in salivary gland was observed in the mice treated with the highest dose of PSMA-1-VcMMAE (3820nmol/kg), indicating no/low toxicity to the gland. Imaging with ^125^I labeled PSMA-1 showed no uptake in salivary gland, which explained no toxicity of PSMA-1-VcMMAE (Figure S11). This will be an advantage as compared to radionuclide therapy. PSMA has also been found in normal prostate, but at significantly lower level as compared to prostate cancers. No adverse histological changes were observed in prostate gland after the treatment with PSMA-1-VcMMAE. Furthermore, MMAE is more potent against rapidly dividing cells and have reduced toxicity to normal cells. Future biodistribution studies are needed to help fully understand the toxicity of PSMA-1-VcMMAE.

PSMA overexpression in prostate cancer is generally correlated with higher PSA levels and Gleason grade. However, prostate cancer is highly heterogeneous, it is known that there are patients with high grade prostate tumors that do not overexpress PSMA. On the other hand, there are also some patients that express high levels of PSMA that do not have aggressive prostate cancer. It will be important to have a full assessment of grade, Gleason score, and PSMA expression of an individual’s cancer before PSMA-1-VcMMAE could be used. Performing PSMA-targeted imaging such as 68Ga-PSMA-617 PET/CT [31] before the treatment will also help the clinician to determine the treatment and predict response to the drug.

## 4. Materials and Methods

### 4.1. General

PSMA targeting peptide Glu-CO-Glu’-Amc-Ahx-Glu-Glu-Glu-Cys-C6-Lys (PSMA-1-Cys-C6-Lys) was synthesized by Fmoc chemistry as previously reported [43]. (S)-2-(3-((S)-5-amino-1-carboxypentyl)ureido)pentanedioic acid (Cys-CO-Glu) was custom made by Bachem Bioscience Inc. (Torrance, CA, USA). All the other chemicals were purchased from Sigma-Aldrich Inc. HPLC was performed on a Shimadzu HPLC system equipped with a SPD-20A prominence UV/visible detector and monitored at 220 and 254 nm. Preparative HPLC was achieved using Luna 5m C18(2) 100A column (250 × 10 × 5 mm; Phenomenex Inc., Torrance, CA, USA) at a flow rate of 2.5 mL/min. Analytical HPLC was performed using an analytical Luna 5 m C18(2) 100A column (250 × 4.6 × 5 mm; Phenomenex) at a flow rate of 0.8 mL/min. The gradient used was 10–90% acetonitrile against 0.1% trifluoroacetic acid over 30 min.

### 4.2. Synthesis of PSMA-Targeting MMAE Conjugates

#### 4.2.1. Synthesis of PSMA-1-VcMMAE

PSMA-1-VcMMAE was synthesized as a prodrug by conjugating MMAE to the Cys residue in PSMA-1-Cys-C6-Lys via a maleimido caproyl valine-citrulline (Vc) cathepsin-cleavable linker with a self-immolative *p*-aminobenzyl carbamate (PABC) spacer. PSMA-1-Cys-C6-Lys (2.6 mg, 2 μmol) was dissolved in 500 μL of phosphate buffer; then, 2.2 μmol of Vc-MMAE (3.0 mg) (BOC Sci.) in 500 µL of DMF was added. The pH of the reaction mixture was adjusted to 8 by triethylamine. The mixture was stirred at room temperature for 1 h, then went through HPLC to get purified PSMA-1-VcMMAE. Yield: 4.4 mg (85%). Retention time: 16.8 min. MS (C_123_H_195_N_23_O_37_S), calculated: 2618.3; found: 1310.7 ([M+2H]/2), 874.1 ([M+3H]/3) (Figure S12).

#### 4.2.2. Synthesis of PSMA-1-VcMMAE-Cy5.5

PSMA-1-Cys-C6-Lys (2.6 mg, 2 μmol) was dissolved in 500 uL of phosphate buffer; then 2.2 μmol of Vc-MMAE (3.0 mg) (BOC Sci.) in 500 µL of DMF was added. The pH of the reaction mixture was adjusted to 8 by trimethylamine. After stirring at room temperature for 1 h, Cy5.5 NHS ester 2.5 μmol in 200 μL of DMF was added. The reaction mixture was stirred overnight, then the product was purified by semipreparative HPLC. Yield: 3.1 mg (50%). Retention time: 18.8 min. MS (C_163_H_236_N_25_O_38_S), calculated: 3185.8; found: 1062.2 ([M+3H]/3), 796.9 ([M+4H]/4) (Figure S12).

#### 4.2.3. Synthesis of PSMA-1-McMMAE-Cy5.5

PSMA-1-McMMAE-Cy5.5 was synthesized using a noncleavable maleimido caproyl (Mc) linker. It was synthesized in the same way as PMA-1-MMAE-Cy5.5. Yield: 45%. Retention time: 20.9 min. MS (C_144_H_209_N_20_O_33_S), calculated: 2780.4; found: 1041.2 ([M+H+Na]/2), 942.2 ([M+H+2Na]/3) (Figure S12).

### 4.3. Cell Culture

Retrovirally transfected PSMA positive PC3pip cells and transfection control PC3flu cells were obtained from Dr. Michel Sadelain in 2000 (Laboratory of Gene Transfer and Gene Expression, Gene Transfer and Somatic Cell Engineering Facility, Memorial-Sloan Kettering Cancer Center, New York, NY, USA). C4-2 cells were from ATCC. The cells were last sorted and checked by Western blot in 2019; no genetic authentication was performed. Cells were maintained in RPMI1640 medium (Invitrogen Inc., Carlsbad, CA, USA) with 2 mM L-glutamine and 10% Fetal Bovine Serum at 37 °C and 5% CO_2_ under a humidified atmosphere.

### 4.4. Competitive Binding Assay

The assay was carried as previously reported [43] by incubation PC3pip cells with different concentrations of drug conjugates in the presence of 10 nM N-[N-[(*S*)-1,3-dicarboxypropyl]carbamoyl]-*S*-[^3^H]-methyl-L-cysteine (^3^H-ZJ24) (GE Healthcare Life Sciences, Chicago, IL, USA). Radioactivity of cell pellet was counted by scintillation counter. The concentration required to inhibit 50% of binding was determined (IC_50_) by GraphPad Prism 3.0.

### 4.5. Enzymatic Cleavage of PSMA-1-MMAE-Cy5.5 by Cathepsin B

PSMA-1-VcMMAE-Cy5.5 or PSMA-1-McMMAE-Cy5.5 was added to 500 μL of activated human liver cathepsin B (Anthens Research and Technology, Anthens, AL, USA) solution [72] to a final concentration of 2 μM and incubated at 37 °C. At different time intervals, 40 μL of the solution was taken out into tubes loaded with 1 μL of 1 mM E64 protease inhibitor. The mixture was vortexed and then stored at −80 °C for future HPLC analysis to assess degradation of PSMA-1-VcMMAE-Cy5.5 or PSMA-1-McMMAE-Cy5.5. Studies were performed in triplicate.

### 4.6. In Vitro Cellular Uptake Studies

PC3pip and PC3flu cells were seeded in µ-Slide 8-Well Chamber Slide (ibidi GmbH, Munich, Germany) at 2000 cells/well. When cells grew to 70% confluency, PSMA-1-VcMMAE-Cy5.5 or PSMA-1-McMMAE-Cy5.5 conjugations were added at 50 nM and incubated at 37 °C for 4 h. Cells were washed with PBS and stained with DAPI and LysoOrange (Abcam Inc., Cambridge, UK) for 30 min at 37 °C and 5% CO_2_, after which they were washed again with PBS and fresh medium added. Selectivity was determined by including 20-fold excess of the PSMA-1 ligand. The uptake and localization of the drug conjugates were visualized under a confocal microscope (Leica Biosystem, Wetzlar, Germany) at 40×.

### 4.7. Immunofluorescence Analysis of Alpha-Tubulin

Cells on coverslips at about 70% confluency were incubated with 5 nM of MMAE, PSMA-1-VcMMAE-Cy5.5, or PSMA-1-McMMAE-Cy5.5 for 24 h. Cells were washed and fixed with 4% paraformaldehyde for 10 min, permeabilized with 0.1% Triton™ X-100 for 10 min, and blocked with 1% BSA for 1 h at room temperature. Alpha-tubulin (B5-1-2) Alexa Fluor 488 Mouse Monoclonal Antibody (Invitrogen) was then added at 2 µg/mL in 0.1% BSA and incubated for 3 h at room temperature. Cells were counterstained with DAPI, mounted with Fluor-Mount aqueous mounting solution, and observed under Leica DM4000B fluorescence microscopy at 40×.

### 4.8. In Vitro Cytotoxicity Assay

Cells (1000/well) were seeded in 96-well culture plates the day before treatment. Cells were incubated with various concentrations of drugs for 72 h and cell viability evaluated by CCK-8 (Dojindo Inc., Rockville, MD, USA). The concentration required to reach 50% of cell proliferation was determined (EC_50_) by GraphPad Prism 3.0.

### 4.9. In Vivo NIR Imaging Studies

Under guidelines of the animal care and use committee at Case Western Reserve University (IACUC#150033) 6–8-week old male Balb/c athymic nude mice were implanted subcutaneously with 1 × 10^6^ of PC3flu and PC3pip in 100 μL of matrigel on the left and right flank, respectively. Mice received 40 nmol/kg of PSMA-1-VcMMAE-Cy5.5 or PSMA-1-McMMAE-Cy5.5 in PBS via tail vein injection when tumors reached 10 mm in diameter. Fluorescence imaging was performed using the Maestro In Vivo Imaging system (Perkin-Elmer Inc., Waltham, MA, USA). At 72-h post injection, mice were euthanized, tumor and organs were extracted for ex vivo imaging. Tumors were snap-frozen in OCT, cut into 10 μm thick sections and fixed on slides. The fluorescence signal from cy5.5 was observed under a Leica DM4000B microscope.

### 4.10. Determination of Maximum Tolerated Dose (MTD)

Groups of three male mice received single injections of MMAE, PSMA-1-Vc-MMAE, or PSMA-ADC [27] via the tail vein to determine single-dose MTD. Mice were monitored daily for 14 days. The MTD was defined as the highest dose that did not cause serious overt toxicities or 20% weight loss in any of the animals.

### 4.11. Heterotopic Survival Study

Male athymic nude mice were implanted subcutaneously with 1 × 10^6^ of PC3pip cells 100 μL of matrigel. When tumor size reached approximately 100 mm^3^ (tumor volume = Length × width^2^/2), mice received drugs through tail vein injection every 4 days with a total of five doses. Mice were treated every 4 days with a total of five doses. Animals were weighed and tumor size measured every other day for 90 days. Cures were defined as no tumor present at end of the 90-day study. When tumors became too large or animals were moribund they were euthanized. Five mice were used in each group.

### 4.12. Orthotopic Survival Study

Surgical orthotopic tumor implantation was performed as previously reported [43]. Tumor growth/size was monitored using Siemens Acuson S2000 (Siemens AG. Munich, Germany) ultrasound scanner. When the tumors were at the appropriate size (5 mm diameter as measured by ultrasound, approximately 2 weeks) animals were given PSMA-1-VcMMAE, or PBS every 4 days with a total of five doses. Tumors were monitored every other day by ultrasound. Each group utilized five mice.

### 4.13. Metastatic Survival Study

Male athymic nude mice were injected into the left ventricle of the heart with 1 × 10^5^ GFP labeled PC3pip cells [73,74] to generate bone metastasis. One week later, mice received 1910 nmol/kg of PSMA-1-VcMMAE or PBS every 4 days with a total of five doses and the progression of disease was monitored by GFP imaging. Eight mice were used in each group.

### 4.14. Statistics

Student’s *t*-test was used to compare inter-group differences. Kaplan–Meier survival data were analyzed by SAS9.4 using log-rank tests. A *p* value < 0.05 was considered statistically significant for all comparisons.

## 5. Conclusions

In summary, we developed a small-molecule-based prodrug for the treatment of prostate cancer. The data demonstrate development of an effective SMDC and the potential utility of this agent for further development. The PSMA-1-VcMMAE was proven to have in vivo efficacy for different tumor cell lines and different mouse models of human prostate cancer, including metastatic disease, with little to no systemic toxicity. Our approach demonstrated an improved therapeutic index compared to either MMAE or PSMA-ADC. PSMA-1-VaMMAE has the promise to improve patients’ overall quality of life, reduce the need for subsequent therapy and do so more cost-effectively. Further studies for dosing optimization, efficacy, and full toxicity studies paving the way for investigational new drug (IND) submission are warranted and supported.

## Figures and Tables

**Table 1:** In vitro cytotoxicity of PSMA-1-MMAE, MMAE, and PSMA-ADC.

Cells	PSMA-1-VcMMAE (EC_50_, nM)	PSMA-1-VcMMAE + PSMA-1 (EC_50_, nM)	PSMA-1-VcMMAE + E64 (EC_50_, nM)	MMAE (EC_50_, nM)	PSMA-ADC (EC_50_, nM)
**PC3pip**	4.64 ± 0.83	241.1 ± 8.6	31.09 ± 2.11	0.24 ± 0.05	0.063 ± 0.003
**PC3flu**	221.7 ± 2.8	261.3 ± 5.6	384.9 ± 5.3	0.21 ± 0.04	241.8 ± 9.3

## Data Availability

Data is contained within the article and supplementary information.

## Supplementary Materials

The following are available online at https://www.mdpi.com/2072-6694/13/3/417/s1. Figure S1. Chromatogram of incubation studies with Cathepsin. Figure S2. Quantification of fluorescent signal on cells treated with PSMA-1-MMAE-Cy5.5 conjugates. Values are mean ± SD of 5 different areas. Figure S3. Caspase 3 studies of PC3pip tumors 4 days after treatment with 160 nmol/kg of PSMA-1-MMAE-Cy5.5 conjugates. Figure S4. Antitumor activity of PSMA-1-VcMMAE-Cy5.5 to mice bearing heterotopic PC3pip tumors. Mice received drug through tail vein injection. Figure S5. Histological examination of major organs treated by 3820 nmol/kg of PSMA-1-VcMMAE. Figure S6. PSMA-VcMMAE (384 nmol/kg), MMAE (700 nmol/kg) and PSMA-ADC (50nmol/kg) showed comparable antitumor activity against heterotopic PC3pip tumors for 30 days. Table S1. Comparison of therapeutic index of PSMA-1-VcMMAE, MMAE and PSMA-ADC. Figure S7. PSMA-1VcMMAE-Cy5.5 is more potent for killing heterotopic PC3pip tumor than for heterotopic PC3flu tumors. Figure S8. PSMA-1-VcMMAE was effective in heterotopic C4-2 tumor model. Figure S9. Establishment of metastatic PC3pipGFP tumor model. Figure S10. Mass spectrum of PSMA-1-VcMMAE, PSMA-1-VcMMAE-Cy5.5 and PSMA-1-McMMAE-Cy5.5. Figure S11. Overlay of gamma scintigraphy of [I-125]-PSMA-1 with planar X-ray. Figure S12. Mass spectrum of PSMA-1-VcMMAE, PSMA-1-VcMMAE-Cy5.5 and PSMA-1-McMMAE-Cy5.5.
