# Supplementary material for: Small Molecule-Based Prodrug Targeting Prostate Specific Membrane Antigen for the Treatment of Prostate Cancer
Source: Cancers (Basel). 2021 Jan 22;13(3):417. doi: 10.3390/cancers13030417 (PMC7865627; doi:10.3390/cancers13030417)
Supplement: Supplementary file 1 [file cancers-13-00417-s001.pdf]

cancers

Supplementary

Small Molecule-Based Prodrug Targeting Prostate Specific Membrane Antigen for the Treatment of Prostate Cancer

Xinning Wang et al.

Supplementary materials

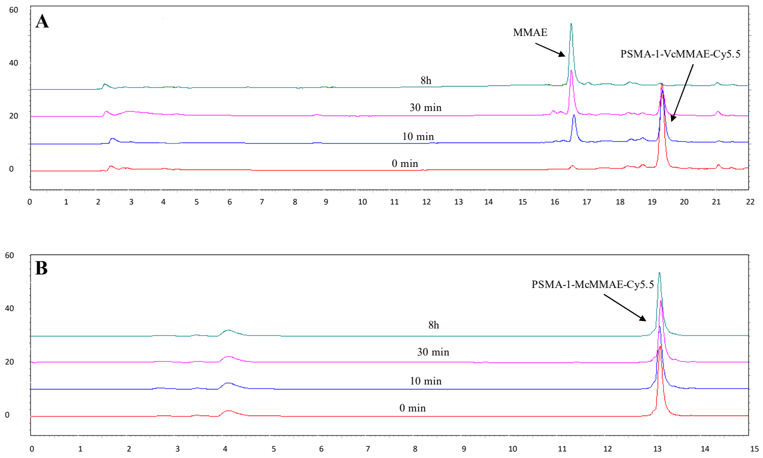

Figure 1. Chromatogram of incubation studies with Cathepsin. (**A**) PSMA-1-VcMMAE-Cy5.5 was degraded and released MMAE when incubated with cathepsin. (**B**) PSMA-1-McMMAE-Cy5.5 remained intact in the presence with cathepsin.

Citation: Wang, X.; Shirke, A.; Walker, E.; Sun, R.; Ramamurthy, G.; Wang, J.; Shan, L.; Mangadlao, J.; Dong, Z.; Li, J.; et al. Small Molecule-Based Prodrug Targeting Prostate Specific Membrane Antigen for the Treatment of Prostate Cancer. *Cancers* **2021**, *13*, 417. https://doi.org/10.3390/cancers13030417

Received: 12 December 2020
Accepted: 19 January 2021
Published: 22 January 2021

Publisher's Note: MDPI stays neutral with regard to jurisdictional claims in published maps and institutional affiliations.

Copyright: © 2021 by the authors. Licensee MDPI, Basel, Switzerland. This article is an open access article distributed under the terms and conditions of the Creative Commons Attribution (CC BY) license (http://creativecommons.org/licenses/by/4.0/).

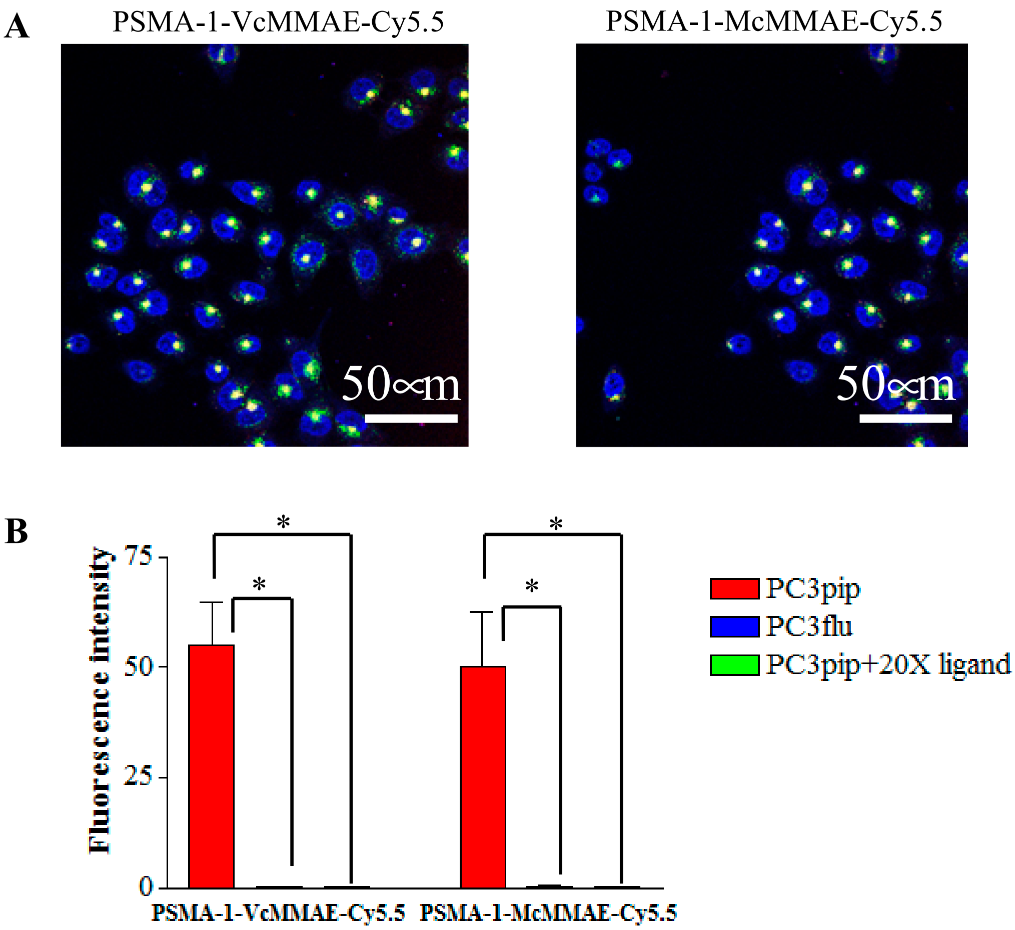

Figure 2. In vitro cellular uptake results of PSMA-targeted drug conjugates. (**A**) Representative fluorescence images of PC3pip cells treated with PSMA-1-VcMMAE-Cy5.5 or PSMA-1-McMMAE-Cy5.5. Nuclei were stained by DAPI and are false colored blue, lysosomes were detected by LysoOrange and are false colored green and drug conjugates are false colored red. (**B**) Quantification of fluorescent signal on cells treated with PSMA-1-MMAE-Cy5.5 conjugates. Values are mean ± SD of 5 different areas. (*: $p < 0.05$).

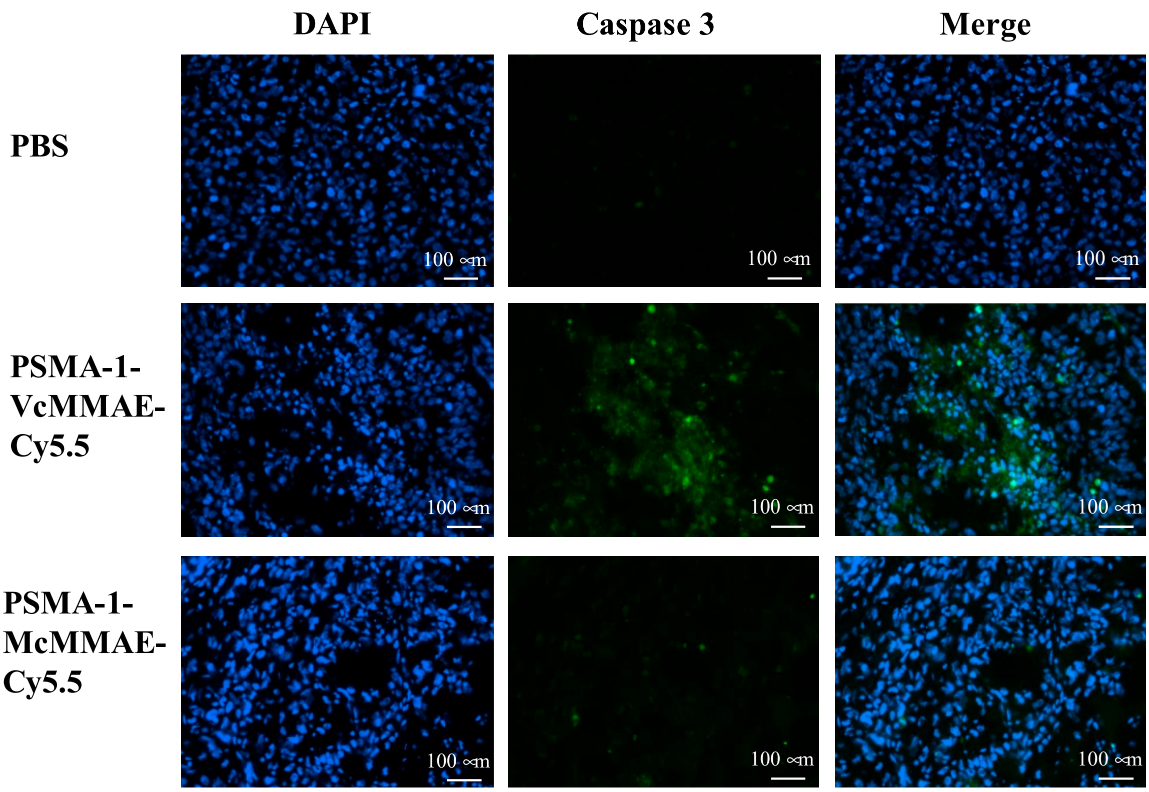

Figure 3. Caspase 3 studies of PC3pip tumors 4 days after treatment with 160 nmol/kg of PSMA-1-MMAE-Cy5.5 conjugates. Tumors were snap-frozen in OCT, cut into 10 µm thick sections and fixed on slides. Induction of apoptosis by the treatment was determined by rabbit polyclonal anti-Caspase-3 antibody. A goat anti-rabbit polyclonal antibody labeled by Alexa Fluor 594 was used as secondary antibody. Fluorescence of DAPI is false colored blue and fluorescence of caspase 3 is false colored green (n = 5).

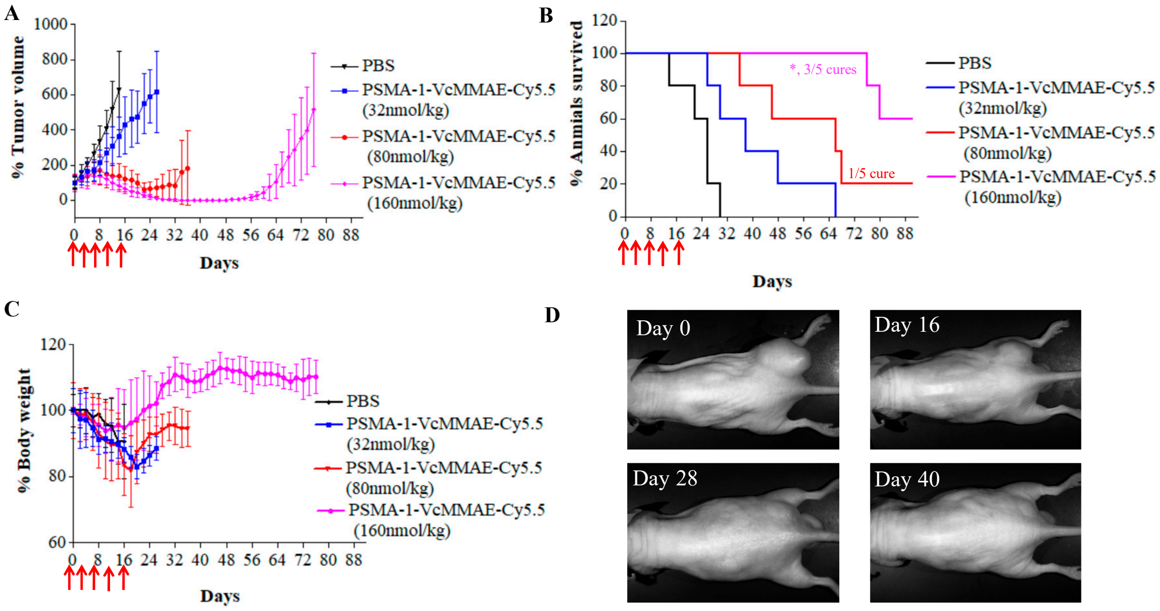

Figure 4. Antitumor activity of PSMA-1-VcMMAE-Cy5.5 to mice bearing heterotopic PC3pip tumors. Mice received drug through tail vein injection. Treatment were scheduled every 4 days with a total of five doses as indicated by the red arrow. Each group had 5 mice. For tumor growth curves and body weight curves, values are mean ±SD of 5 animals. The plots stopped when animals died during the experiments since values are represent as mean ± SD of 5 animals. (**A**) Tumor growth curves of mice treated with PSMA-1-VcMMAE-Cy5.5. (**B**) Kaplan-Meier survival curves of mice treated with PSMA-1-VcMMAE-Cy5.5 (*, PSMA-1-VcMMAE-Cy5.5 160 nmol/kg *vs* PBS, p =0.0004). (**C**) Body weight of mice treated with PSMA-1-VcMMAE-Cy5.5. (**D**) PSMA-1-VcMMAE-Cy5.5 showed the ability to inhibit large tumor (>2000 mm^3) growth (n = 2).

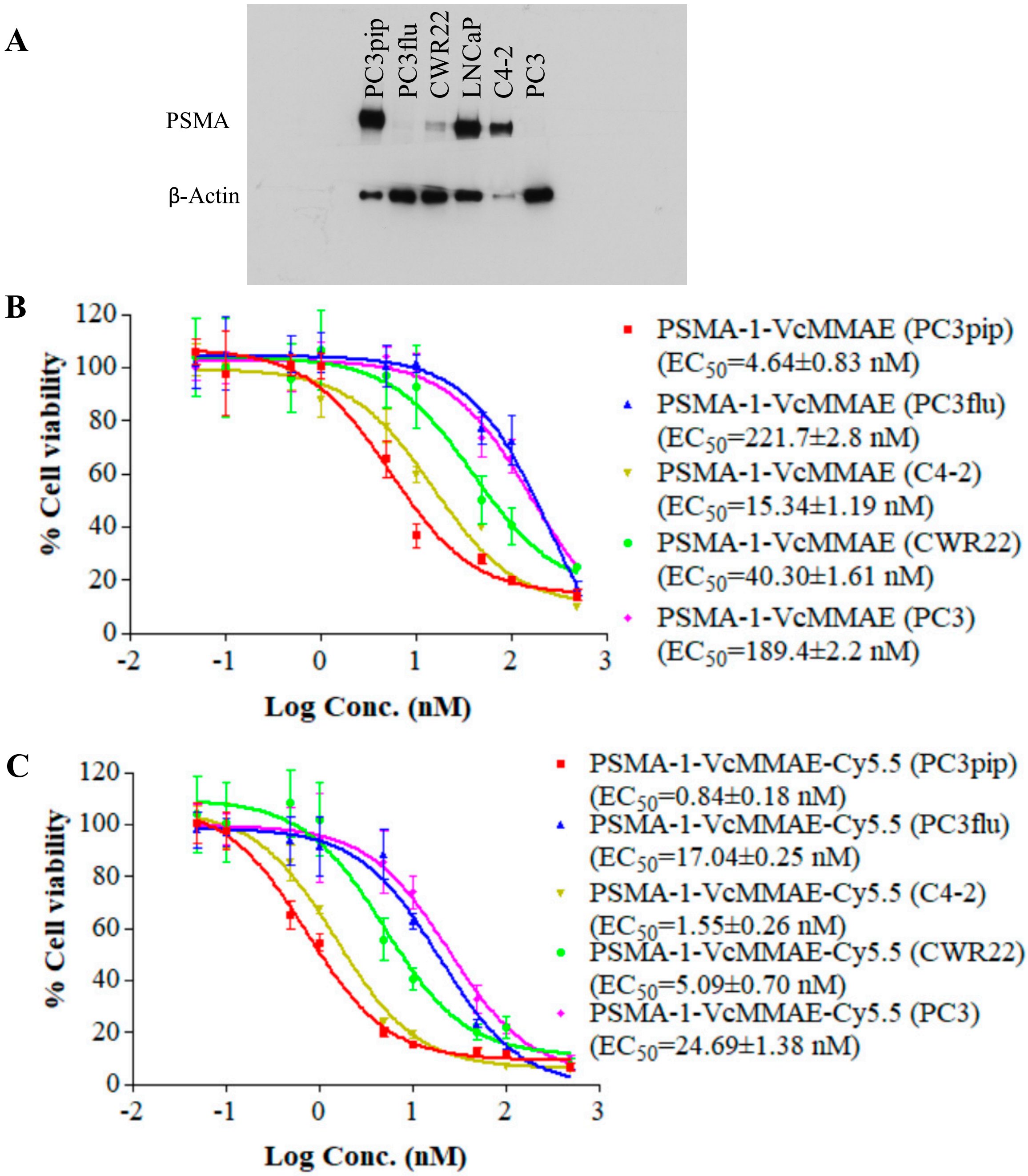

Figure 5. Cytotoxicity of PSMA-1-VcMMAE and PSMA-1-VcMMAE-Cy5.5 to cells with different PSMA expression level. (**A**) Western blot analysis of PSMA expression in different prostate cancer

cells. (**B**) In vitro cytotoxicity of PSMA-1-VcMMAE to different prostate cancer cells. (**C**) In vitro cytotoxicity of PSMA-1-VcMMAE-Cy5.5 to different prostate cancer cells.

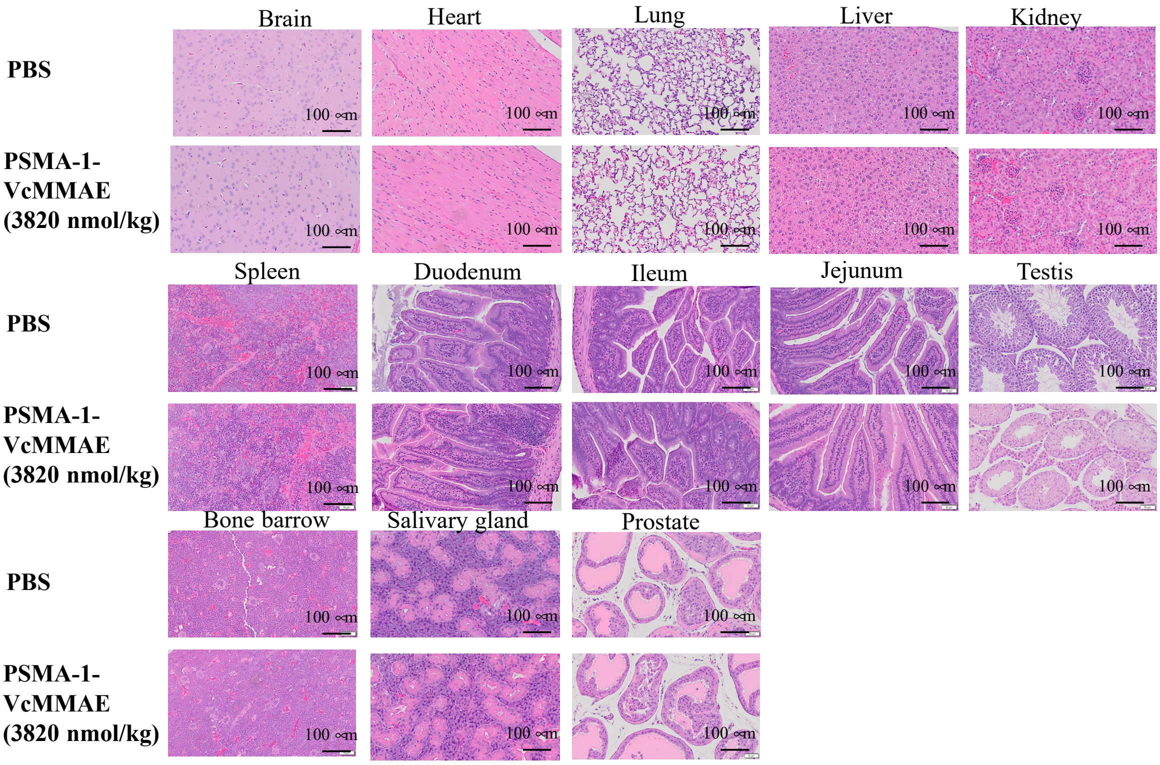

Figure 6. Histological examination of major organs treated by 3820 nmol/kg of PSMA-1-VcMMAE. Mice receive PSMA-1-VcMMAE through tail vein inject every 4 days with total of 5 injections. Mice were sacrificed on day 21 and organs were taken for H&E staining. The slides were read by a pathologist to confirm the histological changes.

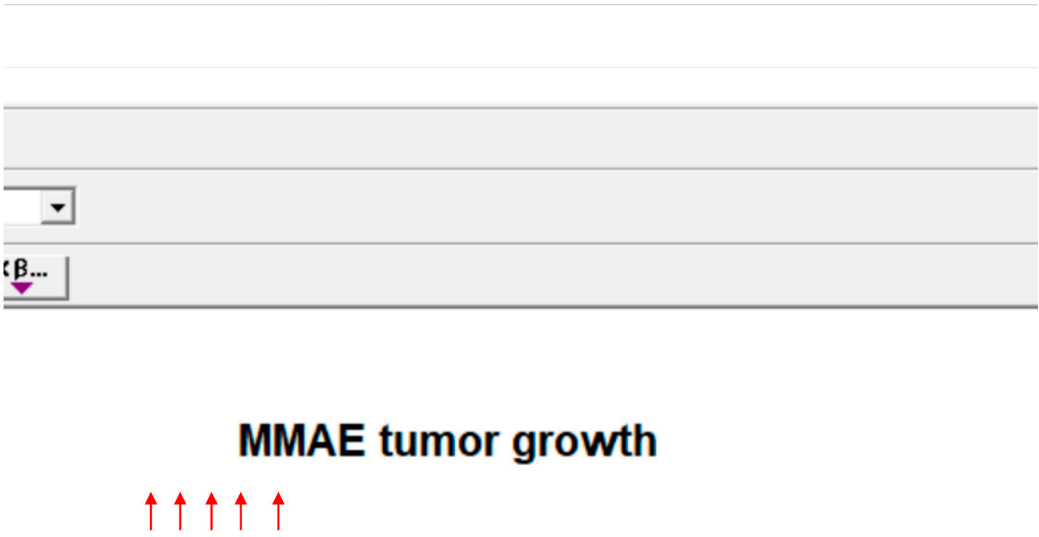

Figure S7. PSMA-VcMMAE (384 nmol/kg), MMAE (700 nmol/kg) and PSMA-ADC (50nmol/kg) showed comparable antitumor activity against heterotopic PC3pip tumors for 30 days. Mice receive

drugs through tail vein inject every 4 days with total of 5 injections as indicated by the red arrows. Each group had 5 mice. Values are mean ± SD of 5 animals. The plots stopped when animals died during the experiments since values are represent as mean ± SD of 5 animals.

Table 1. Comparison of therapeutic index of PSMA-1-VcMMAE, MMAE and PSMA-ADC.

Drugs	MTD (nmol/kg)	Lowest Dose to Effectively Inhibit Tumor Growth (nmol/kg)	Therapeutic Index *
PSMA-1-VcMMAE	7640	382	20
MMAE	700	700	1
PSMA-ADC	640	50	12.8

*: Therapeutic index is defined by MTD/effective dose.

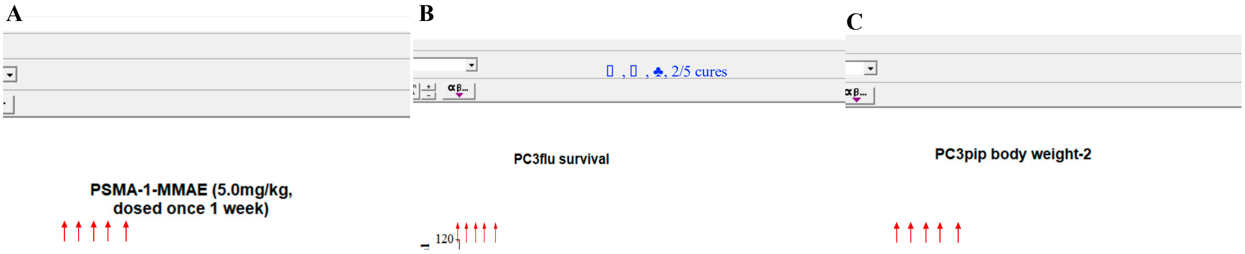

Figure 8. PSMA-1-VcMMAE is more potent for killing heterotopic PC3pip tumor than for heterotopic PC3flu tumors. Treatment were scheduled every 4 days with a total of five doses as indicated by the red arrow. Each group had 5 mice. For tumor growth curves and body weight curves, values are mean ± SD of 5 animals. The plots stopped when animals died during the experiments since values are represent as mean ± SD of 5 animals. (**A**) Tumor growth curves of mice treated with 955 nmol/kg of PSMA-1-VcMMAE. (**B**) Comparison of the potency of PSMA-1-VcMMAE to treat PC3pip (solid line) and PC3flu tumors (dashed line) at the dose of 955 nmol/kg. (☀, PC3pip treated versus PC3pip PBS, $p = 0.0005$; ★, PC3pip treated versus PC3flu PBS, $p = 0.0242$; ♣, PC3pip treated versus PC3flu treated, $p = 0.0493$). (C) Body weight changes of mice treated with 955 nmol/kg of PSMA-1-VcMMAE.

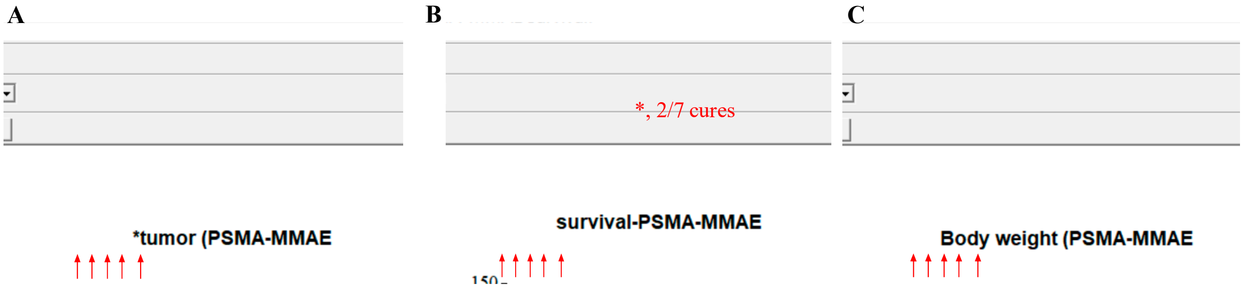

Figure 9. PSMA-1-VcMMAE was effective in heterotopic C4-2 tumor model. Mice bearing heterotopic C4-2 tumor were treated with 1910 nmol/kg of PSMA-1-VcMMAE every 4 days with a total of five doses. Red arrows indicate the time of treatment. For tumor growth curves and body weight curves, values are mean ± SD of 7 animals. The plots stopped when animals died during the experiments since values are represent as mean±SD of 7 animals. (**A**) Tumor growth curves. (**B**) Kaplan-Meier survival curves of mice. (*, PSMA-1-VcMMAE versus PBS, $p = 0.003$); (**C**) Body weight changes of mice.

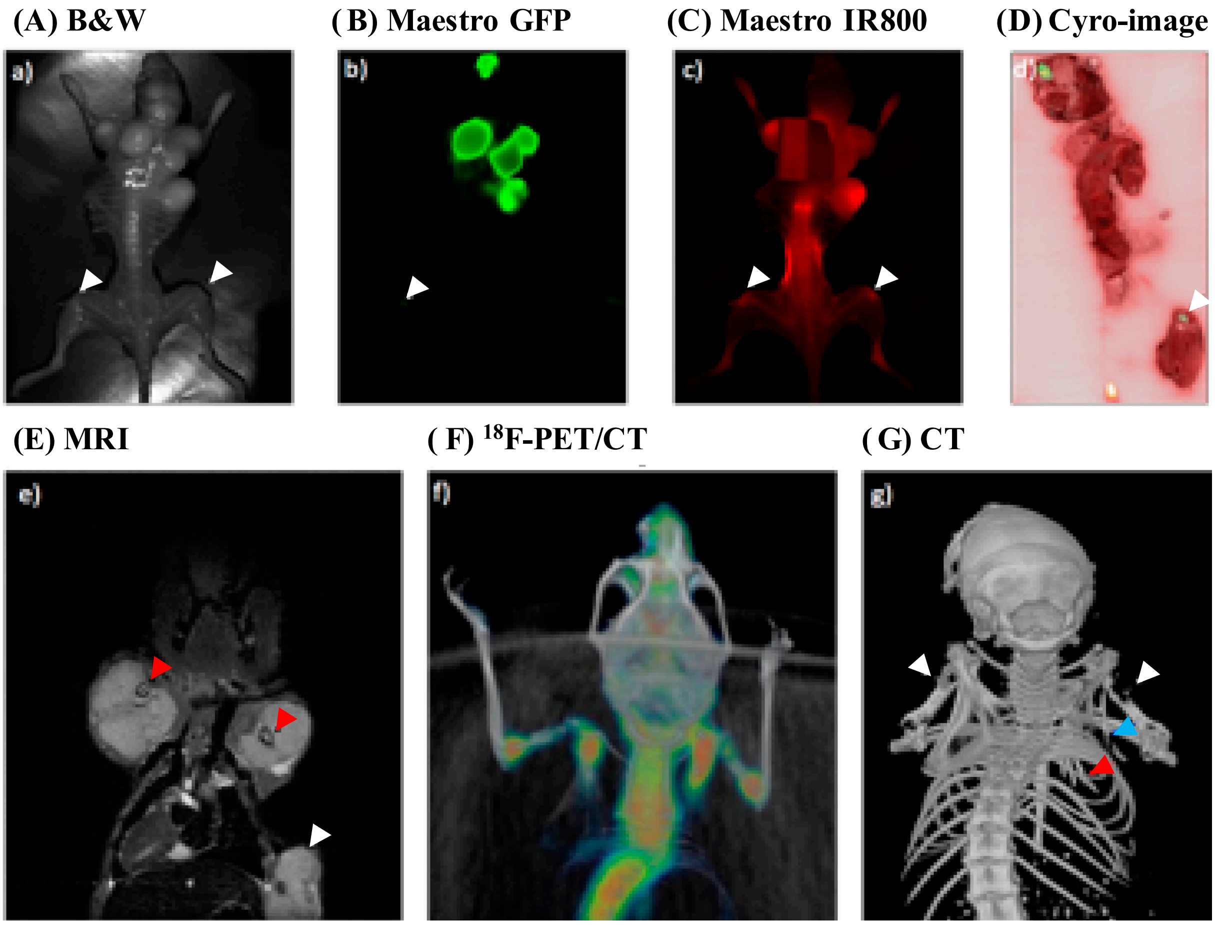

Figure 10. Establishment of metastatic PC3pipGFP tumor model. Four to 6 weeks post tumor inoculation, mice were imaged as indicated to reveal metastatic disease that had migrated from the femurs (earlier times, not shown) and then moved into the spine and shoulders. (**A**) Black and white optical image of skinned mouse; (**B**,**C**) Show the corresponding Maestro fluorescence images using GFP and IR800 channels, respectively. Arrows indicate metastasis in the tibia where both GFP and IR800 fluorescence signals were found. Mice receive PSMA-1-IR800 4 hour before sacrificed. Note, that GFP expression was lost in some tumor masses. (**D**) Fluorescence cryo-section of another mouse shows bright GFP signal in the left tibia (arrow). (**E**) MRI reveals tumors surrounding both left and right humerus (red arrows indicate bone location). The MRI also shows the tumor attached to the rib (white arrow). Finally, PET and CT are indispensable imaging techniques for bone structure analysis. (**F**)^{18}F-PET/CT image showing more uptake in the tumor site which is similar phenomenon observed in patients with prostate cancer. The bright signal in the humerus and the scapula (shoulder blades) as compared to other skeletal parts are indicative of advanced metastasis in the bone. This is further substantiated by a high-resolution CT scan (**G.**) showing broken humerus (white arrows), decayed shoulder blades (blue arrow) and broken rib (red arrow).

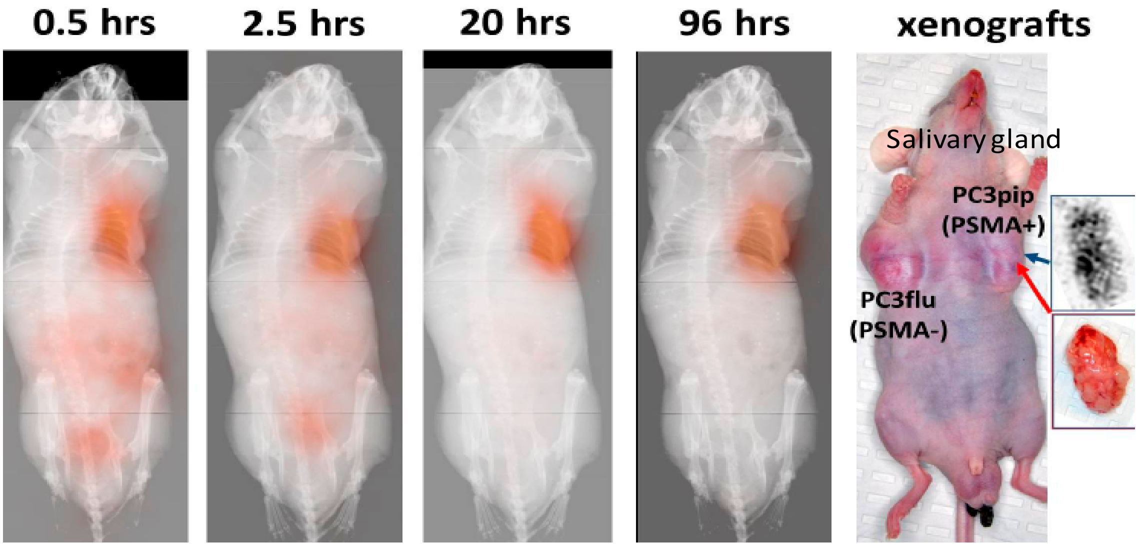

Figure 11. Overlay of gamma scintigraphy of [I-125]-PSMA-1 with planar X-ray. Mouse bearing PSMA-positive PC3pip (right) and PSMA-negative PC3flu (left) tumors were injected with 250 uCi of [I-125]-PSMA-1. 96 hrs post-injection (p.i.), a pin-hole collimator replaced the LEAP collimator for zoomed-in scan (inset) of tumor uptake. No uptake in salivary gland was observed.

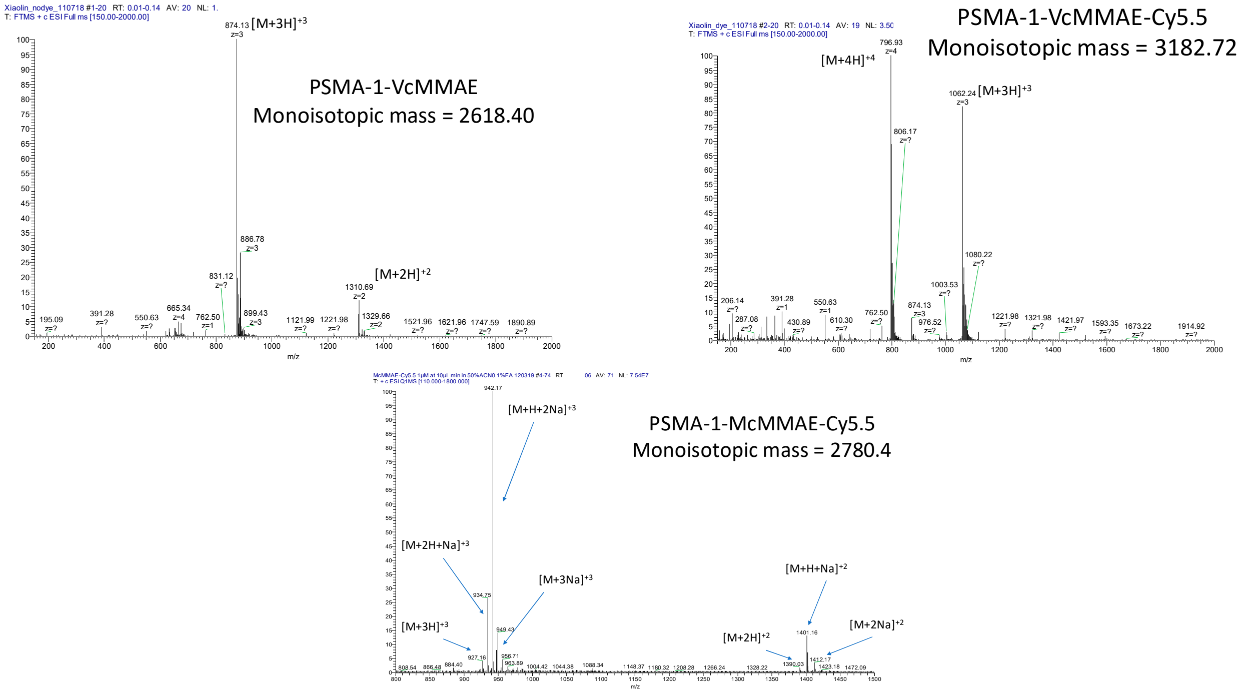

Figure 12. Mass spectrum of PSMA-1-VcMMAE, PSMA-1-VcMMAE-Cy5.5 and PSMA-1-McMMAE-Cy5.5.
